# Multi-Omic Analyses Provide Links between Low-Dose Antibiotic Treatment and Induction of Secondary Metabolism in Burkholderia thailandensis

**DOI:** 10.1128/mBio.03210-19

**Published:** 2020-02-25

**Authors:** Anran Li, Dainan Mao, Aya Yoshimura, Paul C. Rosen, W. Lance Martin, Étienne Gallant, Martin Wühr, Mohammad R. Seyedsayamdost

**Affiliations:** a Department of Molecular Biology, Princeton University, Princeton, New Jersey, USA; b Department of Chemistry, Princeton University, Princeton, New Jersey, USA; c Lewis Sigler Institute for Integrative Genomics, Princeton University, Princeton, New Jersey, USA; California Institute of Technology

**Keywords:** antibiotics, biosynthesis, natural products, secondary metabolism

## Abstract

Low doses of antibiotics can trigger secondary metabolite biosynthesis in bacteria, but the underlying mechanisms are generally unknown. We sought to better understand this phenomenon by studying how the antibiotic trimethoprim activates the synthesis of the virulence factor malleilactone in Burkholderia thailandensis. Using transcriptomics, quantitative multiplexed proteomics, and primary metabolomics, we systematically mapped the changes induced by trimethoprim. Surprisingly, even subinhibitory doses of the antibiotic resulted in broad transcriptional and translational alterations, with ∼8.5% of the transcriptome and ∼5% of the proteome up- or downregulated >4-fold. Follow-up studies with genetic-biochemical experiments showed that the induction of malleilactone synthesis can be sufficiently explained by the accumulation of methionine biosynthetic precursors, notably homoserine, as a result of inhibition of the folate pathway. Homoserine activated the malleilactone gene cluster via the transcriptional regulator MalR and gave rise to a secondary metabolome which was very similar to that generated by trimethoprim. Our work highlights the expansive changes that low-dose trimethoprim induces on bacterial physiology and provides insights into its stimulatory effect on secondary metabolism.

## Abstract (precis)

The discovery of antibiotics ranks among the most significant accomplishments of the last century. Although the targets of nearly all clinical antibiotics are known, our understanding regarding their natural functions and the effects of subinhibitory concentrations is in its infancy. Stimulatory rather than inhibitory functions have been attributed to low-dose antibiotics. Among these, we previously found that antibiotics activate silent biosynthetic genes and thereby enhance the metabolic output of bacteria. The regulatory circuits underlying this phenomenon are unknown. We take a first step toward elucidating these circuits and show that low doses of trimethoprim (Tmp) have cell-wide effects on the saprophyte Burkholderia thailandensis. Most importantly, inhibition of one-carbon metabolic processes by Tmp leads to an accumulation of homoserine, which induces the production of an otherwise silent cytotoxin via a LuxR-type transcriptional regulator. These results provide a starting point for uncovering the molecular basis of the hormetic effects of antibiotics.

## INTRODUCTION

The effects of antibiotics on bacteria depend on concentration. While high doses of antibiotics kill, low doses can act more subtly (1–4). Low doses of erythromycin and rifampin, for example, can significantly change the expression levels of 5% of the transcripts in Salmonella enterica serovar Typhimurium, in many cases stimulating gene expression. Understanding better the capacity of low-dose antibiotics to act as signaling molecules or cues will enable new ways to interrogate bacterial signal transduction pathways and guide the improvement of antibiotic therapies (4, 5).

The activation of bacterial secondary metabolism is an emerging dimension of the idea that antibiotics can function as signals (6–9). Secondary metabolites are generated by dedicated biosynthetic gene clusters (BGCs), and aside from carrying out an array of functions for the host, they have also provided a fruitful source of pharmaceutical compounds (10). Prolific bacterial producers may contain >20 BGCs and thus a capacity to produce >20 secondary metabolites. However, most BGCs that can be identified bioinformatically in a given bacterial genome are silent or cryptic, meaning that they are not expressed, weakly expressed, or posttranscriptionally silenced under standard laboratory growth conditions. As a result, the small-molecule products may be synthesized at levels too low to be detected, if they are synthesized at all (11–14).

**FIG 1:**
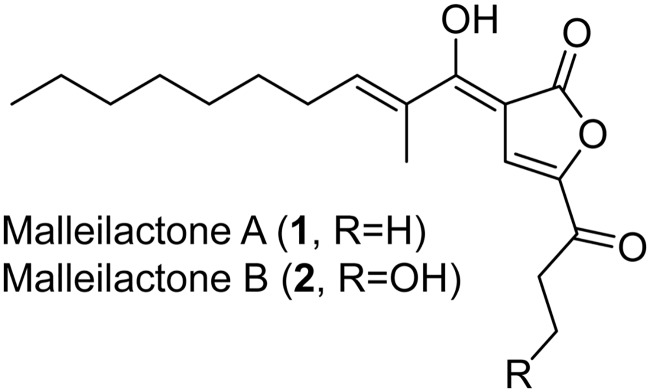
Structures of the cryptic virulence factors malleilactone A and B.

We recently used a chemical-genetic screen to show that low-dose (30 μM) trimethoprim (Tmp), a clinically used antibiotic that inhibits dihydrofolate reductase (DHFR), activates the silent *mal* BGC, which produces the virulence factor malleilactone (also known as burkholderic acid) in Burkholderia thailandensis E264 (Fig. 1) (7, 15–17). We focused on this emerging, saprophytic model organism because it harbors a large cryptic secondary metabolome and is related to the bioterrorism agents Burkholderia mallei and Burkholderia pseudomallei (12, 18). We subsequently used metabolomic approaches to show that Tmp not only induces the *mal* BGC but also broadly activates secondary metabolism in B. thailandensis, leading to the overproduction of 4-hydroxy-3-methylquinolines (HMQs), thailandamides, bactobolins, and capistruins, and to the synthesis of new hybrid metabolites, such as acybolins (19–24).

Several questions remain about Tmp and how it acts as a signal to awaken silent gene clusters. A basic one is, what are the consequences of low-dose Tmp treatment? While the detailed effects of inhibitory levels of Tmp have been characterized, especially in Escherichia coli, they are unknown in B. thailandensis (25–31). The effects of subinhibitory doses of Tmp have not been examined in either bacterium. Another question is, how does Tmp affect BGC expression globally? We have previously mapped the secondary metabolic effects of Tmp, but the cell-wide effects of the antibiotic on the transcriptome and proteome of B. thailandensis are yet to be determined. Perhaps the most pertinent outstanding question is, what connects Tmp with secondary metabolism? The study of secondary metabolic regulation in *Burkholderia* spp. has largely focused on quorum sensing (QS) and transcriptional regulators that intersect with QS circuits. The Greenberg lab previously defined the regulon of the three *N*-acylhomoserine lactone (AHL) systems, demonstrating a complex, intertwined QS network (32–35). The effects of an orphan LuxR (MalR), as well as LysR-type (ScmR) and MarR-type (MftR) transcriptional regulators, have been characterized as well (36–39). However, our understanding of the regulation of silent BGCs in response to exogenous signals is in its infancy. In collaboration with the Greenberg and Chandler groups, we previously reported that the inhibition of DHFR is required for the Tmp-dependent upregulation of the *mal* BGC (36). Whether this applies to how Tmp activates other BGCs and which downstream effectors connect the inhibition of folate biogenesis to secondary metabolism remains unknown.

Herein, we map the global effects of Tmp on B. thailandensis and find that low doses of the antibiotic have a significant, cell-wide impact on its transcriptome and proteome. We find that cellular stress responses are not required for the effect of Tmp and establish a link between Tmp treatment and increased levels of the Met biosynthetic intermediate homoserine, which accumulates in the presence of the antibiotic. The enhanced levels of homoserine are sufficient for induction of the *mal* cluster via the transcriptional regulator MalR. These findings set the stage for understanding the hormetic effects of Tmp and for identifying the molecular components downstream of the Met biosynthetic pathway relevant to secondary metabolism in B. thailandensis.

## RESULTS AND DISCUSSION

### Timing of malleilactone biosynthesis.

We first used a translational *malA-lacZ* reporter to define the time frame of *mal* induction and the impact of low-dose Tmp (30 μM, 5-fold less than the MIC) on the growth of B. thailandensis (see Table S1 in the supplemental material) (40). In agreement with our prior work, Tmp maximally activated *malA* expression at 12 h and an optical density at 600 nm (OD_600_) of ∼1.0 (Fig. S1) (36). Although low-dose Tmp delayed growth slightly, it did not diminish cell density in stationary phase. We reasoned that understanding the effects of Tmp on gene expression at an OD_600_ of ∼1.0 (12 h for Tmp-treated cells) would afford insights into the activation of secondary metabolism, and we conducted whole-cell transcriptomics, proteomics, and metabolomics to generate hypotheses for the stimulatory effects of the antibiotic.

10.1128/mBio.03210-19.1 FIG S1 Growth curve and timing of *mal* upregulation. (A) Growth curve of untreated (black circles) or Tmp-treated (red) wt B. thailandensis cultures. RNA-seq and proteomics analyses were conducted at an OD_600_ of 1 for both DMSO-treated (control) and Tmp-treated cultures. (B) Time course of *mal* upregulation in a *malA-lacZ* reporter strain as a function of Tmp. Optimal induction occurs at 12 h. OD_600_-normalized LacZ activity is shown for both untreated (black) and Tmp-treated (red) cultures. (C) Time course of malleilactone production, determined by quantitative HPLC-MS using integration of the unique 380-nm signal of the natural product. The data are normalized to the 22-h time point (+Tmp), which gave the highest levels of malleilactone. No malleilactone was detected at the 14-h time point. The results of a single replicate are shown. The average of three independent measurements is shown at each time point in panels A and B. Error bars represent the standard error of the mean (SEM). Download FIG S1, PDF file, 0.3 MB. Copyright © 2020 Li et al. 2020 Li et al. This content is distributed under the terms of the Creative Commons Attribution 4.0 International license.

10.1128/mBio.03210-19.5 TABLE S1 Bacterial strains and plasmids generated or used in this study. Download Table S1, DOCX file, 0.1 MB. Copyright © 2020 Li et al. 2020 Li et al. This content is distributed under the terms of the Creative Commons Attribution 4.0 International license.

### Low-dose Tmp controls a regulon of ∼500 genes.

The effect of Tmp on the secondary metabolome of B. thailandensis has previously been explored. However, its impact on the transcriptome and proteome has not yet been determined. To do so, we first used RNA sequencing (RNA-seq) to probe global transcriptional changes relative to an OD_600_-matched dimethyl sulfoxide (DMSO) control. Three biological replicates of each condition were grown to an OD_600_ of ∼1.0, and total RNA was isolated and subjected to RNA-seq. A total of ∼37 million reads were obtained for each replicate. These were analyzed by determining differential gene expression levels for all transcripts mapped onto the B. thailandensis genome. Four hundred ninety-seven genes (of 5,727 total) were upregulated or downregulated at least 4-fold, suggesting an expansive effect of low doses of Tmp on B. thailandensis (Table S2).

10.1128/mBio.03210-19.6 TABLE S2 All genes upregulated (top) or downregulated (bottom) ≥5-fold in response to Tmp as determined by RNA-seq at an OD_600_ of ∼1.0. The averages of three independent measurements are shown. Standard errors were typically <10% of the mean values reported. Download Table S2, DOCX file, 0.1 MB. Copyright © 2020 Li et al. 2020 Li et al. This content is distributed under the terms of the Creative Commons Attribution 4.0 International license.

We complemented the RNA-seq data with a modified version of the tandem mass tag-complement reporter ion approach (TMTc+) for global proteomic analysis. TMTc+ is an emerging proteomic method with improved accuracy and sensitivity for multiplexed detection of protein fragments (41). We collected full OD_600_-matched data sets at an OD of ∼1.0 (12 h for Tmp-treated cells) and an OD_600_ of ∼5 (24 h for Tmp-treated cells) to characterize proteomic changes relative to a DMSO control. We detected 2,463 proteins (43% coverage of the B. thailandensis proteome) at an OD_600_ of ∼1.0 and 2,524 proteins (45% coverage) at an OD_600_ of ∼5.0 (Tables S3 and S4). The extent of this proteome coverage significantly surpasses that achieved in prior proteomic studies of *Burkholderia* spp. (42).

10.1128/mBio.03210-19.7 TABLE S3 Top 25 most upregulated proteins (top) and most downregulated proteins (bottom) as determined by TMTc+ at an OD_600_ of ∼1.0. The averages of three independent measurements are shown. Standard errors were typically <10% of the mean values reported. Download Table S3, DOCX file, 0.1 MB. Copyright © 2020 Li et al. 2020 Li et al. This content is distributed under the terms of the Creative Commons Attribution 4.0 International license.

A Pearson correlation plot comparing the RNA-seq and proteomic data demonstrated that the transcriptional and translational fold changes of all proteins detected at an OD_600_ of ∼1.0 were modestly correlated (*r* = 0.64), suggesting that our RNA-seq and TMTc+ experiments captured, at least partially, a common set of biological features (Fig. S2). One explanation for the modest correlation between the two data sets is that proteins often persist much longer than does mRNA (43). Alternatively, the expression of some proteins may be regulated posttranscriptionally via translational efficiencies or protein degradation rates.

10.1128/mBio.03210-19.2 FIG S2 Transcriptomic and proteomic analyses. (A) Pearson analysis relating Tmp-induced changes in transcript and protein levels for all genes detected at an OD_600_ of ∼1.0. The dotted lines mark the ca. 3-fold and −3-fold thresholds in both axes. (B to D) Annotation of groups of genes most represented in clusters 2 (B), 3 (C), and 5 (D). Fold enrichment indicates the proportion of genes in the annotated category relative to the genome of B. thailandensis. The bubble size indicates the number of genes in each category. Download FIG S2, PDF file, 0.5 MB. Copyright © 2020 Li et al. 2020 Li et al. This content is distributed under the terms of the Creative Commons Attribution 4.0 International license.

### Targeted and unbiased omics analysis.

**FIG 2:**
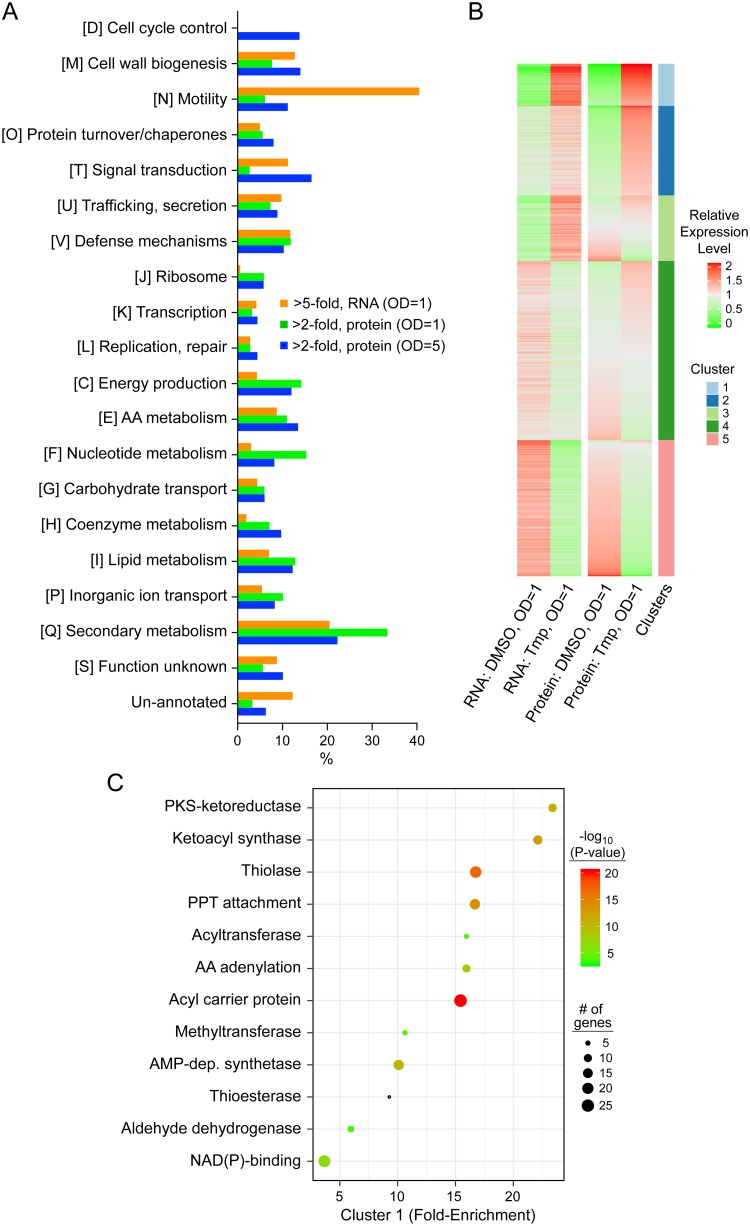
Targeted and unbiased analyses of transcriptomic and proteomic results. (A) Transcriptional and translational effects of low-dose Tmp on the physiological pathways shown (COG functional codes are in brackets followed by definition). The percentages of proteins, the expression of which was altered by ≥2-fold (proteomics) or ≥5-fold (transcriptomics) in response to Tmp within a given pathway, are shown by the bars. (B) Function-agnostic K-means clustering organizes expression changes of all genes/proteins upon Tmp treatment into 5 clusters, which are color coded. The proteins contained within each cluster (other than cluster 4) are annotated in bubble plots (panel C and Fig. S2). (C) Functional enrichment analysis for cluster 1. Fold enrichment indicates the proportion of genes in the category indicated relative to the genome of B. thailandensis. The bubble size indicates the number of genes in each category. AA, amino acid; PKS, polyketide synthase; dep., dependent; PPT, phosphopantetheine.

To analyze the omics data, we took three approaches. We generated differential gene expression tables and visually inspected the most up- and downregulated genes/proteins (Tables S2 to S4). We also conducted a targeted inquiry into the major pathways that were affected by Tmp. Using the eggNOG-mapper, we carried out functional annotation of the B. thailandensis proteome to assign each protein to a Cluster of Orthologous Groups (COG) category (44). The effect of Tmp on these COGs was visualized by plotting the fraction of proteins within a category affected ≥2-fold (proteomics) or ≥5-fold (transcriptomics) in our data sets (Fig. 2A). The results provide a picture of the global effects of Tmp on diverse cellular pathways.

10.1128/mBio.03210-19.3 FIG S3 Tmp targets *dhfr* to induce secondary metabolite production. (A) Secondary metabolite production monitored by HPLC-MS in wt B. thailandensis cultures treated with (red trace) or without (black) Tmp, and in cultures expressing a Tmp-resistant DHFR (*dhfr^r^*) treated with (dark blue) or without (light blue) Tmp. The Tmp-resistant *dhfr^r^* strain fails to produce malleilactone (1) as well as other secondary metabolites. (B) Secondary metabolite production monitored by HPLC-MS in wt B. thailandensis cultures treated with (red trace) or without (black) Tmp, and in the B. thailandensis Δ*relA* mutant treated with (dark blue) or without (light blue) Tmp. The Δ*relA* mutant strain can still produce malleilactone (1) as well as other secondary metabolites. Note that the HMQs (marked with an arrow) are overproduced in untreated Δ*relA* cultures relative to the untreated wt. (C) Secondary metabolite production monitored by HPLC-MS in B. thailandensis cultures that were untreated (black trace), supplemented with Tmp (red), or two different concentrations of Ser hydroxamate (SHX, light and dark blue). No malleilactone was observed upon SHX treatment. A higher concentration of SHX did not elicit malleilactone either (not shown). The peak corresponding to malleilactone (1) is labeled. Download FIG S3, PDF file, 0.3 MB. Copyright © 2020 Li et al. 2020 Li et al. This content is distributed under the terms of the Creative Commons Attribution 4.0 International license.

Last, we paired this targeted analysis with an unbiased search, that is, independent of gene function/annotation. Normalized RNA and protein expression levels were grouped via K-means clustering, leading to the identification of 5 main clusters (Fig. 2B). Cluster 1 consisted of genes/proteins which were significantly upregulated both in the RNA-seq and proteomics data sets, while cluster 5 contained genes/proteins that were significantly downregulated in both sets. Cluster 4, the largest of the five, consisted of genes that did not change significantly in both cases. Clusters 2 and 3 corresponded to genes that were significantly upregulated in one data set (e.g., proteomics) and mildly in the other (e.g., transcriptomics). With the genes clustered in this function-agnostic fashion, functional enrichment analysis was carried out with GO term, InterPro, or KEGG pathway annotation via the DAVID algorithm (45). The results for cluster 1 and for clusters 2, 3, and 5 are shown in Fig. 2C and S2, respectively.

Inspection of the results by the targeted approach showed that the genes involved in secondary metabolism and (to a lesser degree) cell motility, were among the most affected categories (Fig. 2A). The untargeted approach was largely congruent with these findings. Cluster 1 consisted entirely of enzymes involved in secondary metabolism, a finding that is consistent with the observed metabolite output upon low-dose Tmp treatment (Fig. 2C). Cluster 2 revealed enzymes involved in amino acid synthesis and modification. Visual inspection of the transcriptomic and proteomic data sets showed that some flagellar proteins, MetE (a vitamin B_12_-independent methionine biosynthetic enzyme), and again, secondary metabolite biosynthesis proteins were among the most upregulated (Table S2). Cluster 5, which contains genes/proteins downregulated in response to Tmp, was enriched in enzymes involved in translation (Fig. S2). Visual inspection revealed universal stress response proteins, arginine catabolism, and other flagellar proteins as most downregulated. We conclude from these data sets that secondary metabolism is the physiological pathway impacted most by low-dose Tmp.

### Low-dose Tmp enhances intracellular levels of amino acids and NTPs.

**FIG 3:**
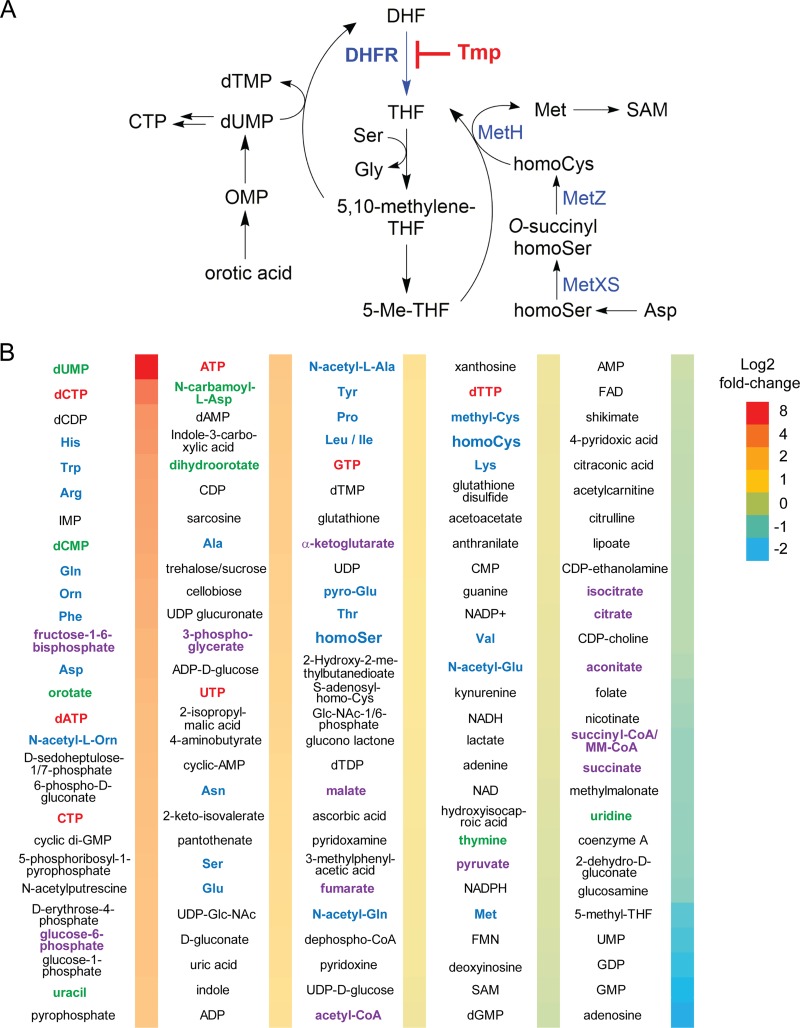
Global primary metabolite analysis upon Tmp treatment in B. thailandensis. (A) Inhibition of DHFR by Tmp affects one-carbon metabolism which is important for the synthesis of Gly, Met, thymidine, SAM, and purines (not shown). (B) Fold change in the level of primary metabolites relative to a DMSO control as determined by quantitative HPLC-MS analysis. Three independent biological replicates for each condition were used. The color coding is as follows: pyrimidine nucleotide biosynthetic intermediates, green; NTPs, red; amino acids, blue; and glycolytic and TCA cycle intermediates, purple. OMP, orotidine 5′-monophosphate; CoA, coenzyme A; FMN, flavin mononucleotide; FAD, flavin adenine dinucleotide; MM, methyl malonyl.

Before following the leads uncovered in the profiling experiments described above, we supplemented the transcriptomic and proteomic data by identifying the effects of low-dose Tmp on the global primary metabolome of B. thailandensis. Tmp targets a critical axis of one-carbon metabolism, an overview of which is provided in Fig. 3A. Previous studies conducted in minimal medium with E. coli showed that inhibitory levels of Tmp result in an accumulation of dUMP, oxidized folates, and most amino acids, while the levels of nucleoside triphosphates (NTPs), reduced folates, Met, *S*-adenosylmethionine (SAM), Ser, and Gly were diminished (30, 31). In line with these studies, comparative OD-matched primary metabolomic analysis revealed enhanced levels of dUMP and orotic acid relative to DMSO-treated B. thailandensis cultures (Fig. 3B). These intermediates likely accumulate as the cell is unable to convert dUMP to dTMP, a process that requires 5,10-methylene-tetrahydrofolate. The concentrations of most amino acids were enhanced as well, including those of the Met biosynthetic precursors homocysteine (homoCys) and homoserine (homoSer). In contrast to studies in E. coli, we found mildly elevated levels of NTPs (Fig. 3B). On the other hand, the levels of Met and SAM were unchanged. These results indicate that low-dose Tmp leads to a physiological state with enhanced intracellular NTPs and amino acids but a diminished one-carbon metabolite pool. The metabolic response that we detect may be homeostatic, since metabolites like 5-Me-THF, Met, and SAM, which Tmp normally suppresses, are only weakly downregulated or are unchanged. Alternatively, the homeostatic levels of Met may be a consequence of the partial inhibition of DHFR by subinhibitory doses of Tmp (see below).

Examination of the omics results led us to assess (i) the effect of low-dose Tmp on cell-wide expression of secondary metabolite BGCs, as these were among the most activated in our data sets (Fig. 2A and C), (ii) the role of previously characterized global regulators of secondary metabolism, *scmR* and *mftR*, (iii) the effect of Tmp on DHFR and the cellular stress response, which was affected upon Tmp addition (Fig. S2), and (iv) the long-range effects of Tmp on Met biogenesis in one-carbon metabolism to examine the role of accumulated intermediates (Fig. 3).

**FIG 4:**
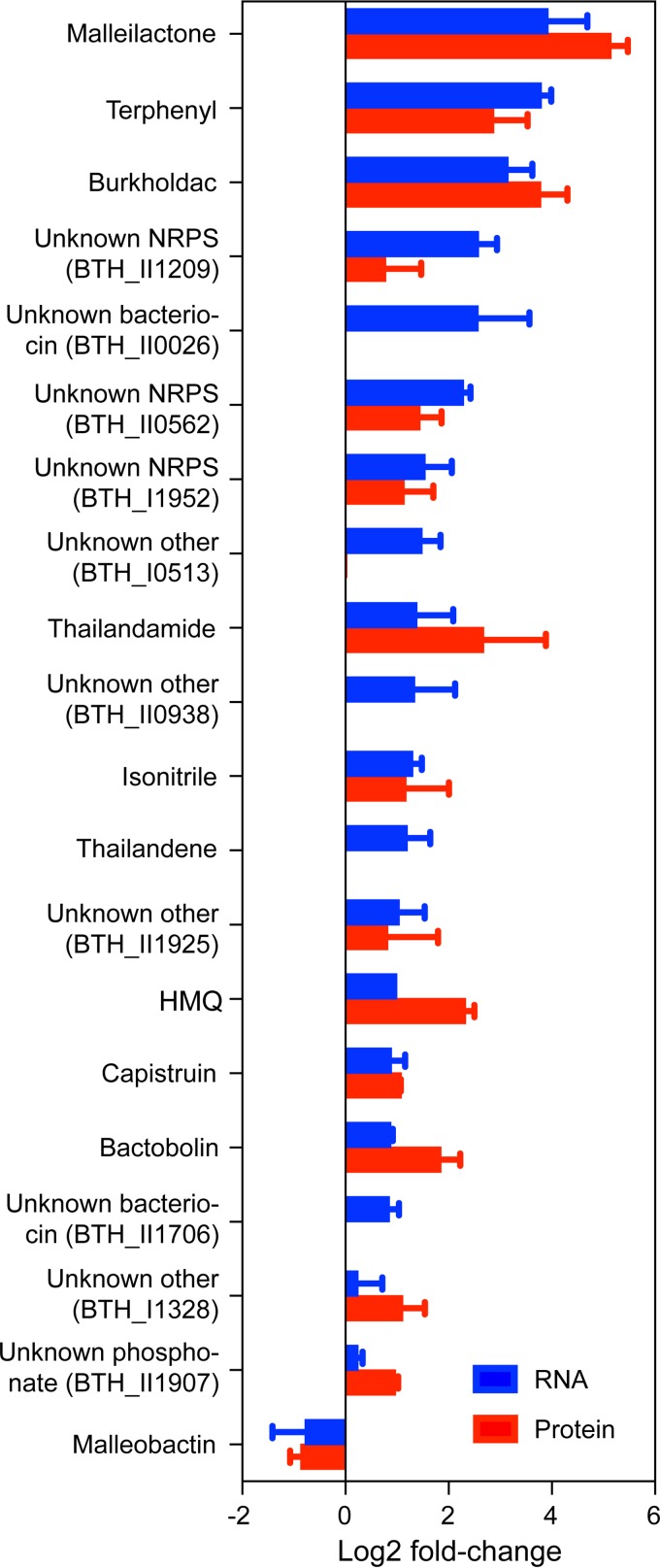
Effect of low-dose Tmp on BGCs in B. thailandensis. Blue and red bars correspond to observed changes in the RNA-seq and TMTc+ data sets at an OD_600_ of ∼1.0, respectively, relative to OD-matched DMSO controls. Changes in the expression of all genes within a cluster were averaged to obtain the values shown. Not all biosynthetic proteins were detected in our TMTc+ data set. Error bars represent standard error of the mean (SEM) from three independent biological replicates. NRPS, nonribosomal peptide synthetase.

**(i) Tmp broadly affects expression of secondary metabolite BGCs.** Using our transcriptomic and proteomic data, we evaluated the cell-wide effects of Tmp on secondary metabolism. Remarkably, with the exception of the malleobactin siderophore cluster, all BGCs were positively regulated with the *mal*, *terphenyl*, and *bhc* BGCs among the most induced both transcriptionally and translationally. At an OD_600_ of ∼1.0, they were induced 15.1 ± 1.7-fold (*mal*), 13.7 ± 1.1-fold (*terphenyl*), and 8.8 ± 1.4-fold (*bhc*), as determined by RNA-seq (Fig. 4). Congruous results were obtained by TMTc+ at an OD_600_ of ∼1.0, where *mal*, *terphenyl*, and *bhc* were induced 35.0 ± 1.3-fold, 7.2 ± 1.6-fold, and 13.6 ± 1.4-fold, respectively. Also upregulated were the *tha*, *hmq*, and *cap* BGCs, along with a number of BGCs with yet-unknown products. These results could enable the identification of their cognate cryptic products in the future. Moreover, they provide the first systematic mapping of how Tmp affects BGC expression levels in B. thailandensis, revealing it to be a cell-wide activator of BGC expression.

**FIG 5:**
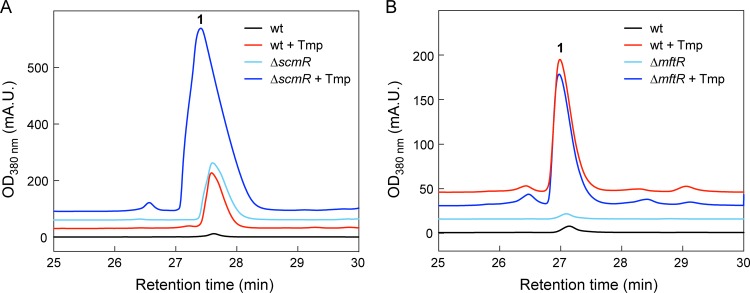
HPLC-MS analysis of B. thailandensis Δ*scmR* and Δ*mftR* mutant cultures grown in LB-MOPS. (A) Malleilactone production in Δ*scmR* mutant cultures treated with (dark-blue trace) or without (light blue) Tmp. (B) Malleilactone production in Δ*mftR* mutant cultures treated with (dark-blue trace) or without (light blue) Tmp. In both panels, the response of wt B. thailandensis is shown for comparison. mA.U., milliabsorbance units.

**(ii) Role of global regulators of secondary metabolism.** We next focused on the LysR-type regulator ScmR and the MarR-type regulator MftR, both of which were downregulated transcriptionally (1.4-fold and 1.6-fold, respectively) and have been shown to suppress secondary metabolism (37, 38). Tmp treatment of a markerless Δ*scmR* mutant strain (Table S1), which constitutively produces malleilactone, resulted in even higher levels of malleilactone, suggesting that the *scmR* and Tmp-mediated pathways for malleilactone induction are independent (Fig. 5A). In our hands, an *mftR* deletion, generated using kanamycin resistance (Kan^r^) insertional mutagenesis (Tables S1 and S5), did not produce malleilactone, in contrast to a previous study in which a Campbell insertion was used to inactivate *mftR* (38). Regardless, the *mftR::kan^r^* strain still produced malleilactone in response to Tmp, suggesting that MftR is not required (Fig. 5B). We conclude that Tmp-induced malleilactone biosynthesis involves factors beyond repression of the known secondary metabolite regulators ScmR and MftR.

10.1128/mBio.03210-19.8 TABLE S4 Top 25 most upregulated proteins (top) and downregulated proteins (bottom) by TMTc+ at an OD_600_ of ∼5.0. The averages of three independent measurements are shown. Standard errors were typically <10% of the mean values reported. Download Table S4, DOCX file, 0.1 MB. Copyright © 2020 Li et al. 2020 Li et al. This content is distributed under the terms of the Creative Commons Attribution 4.0 International license.

10.1128/mBio.03210-19.9 TABLE S5 Primers used in this study. Download Table S5, DOCX file, 0.1 MB. Copyright © 2020 Li et al. 2020 Li et al. This content is distributed under the terms of the Creative Commons Attribution 4.0 International license.

**FIG 6:**
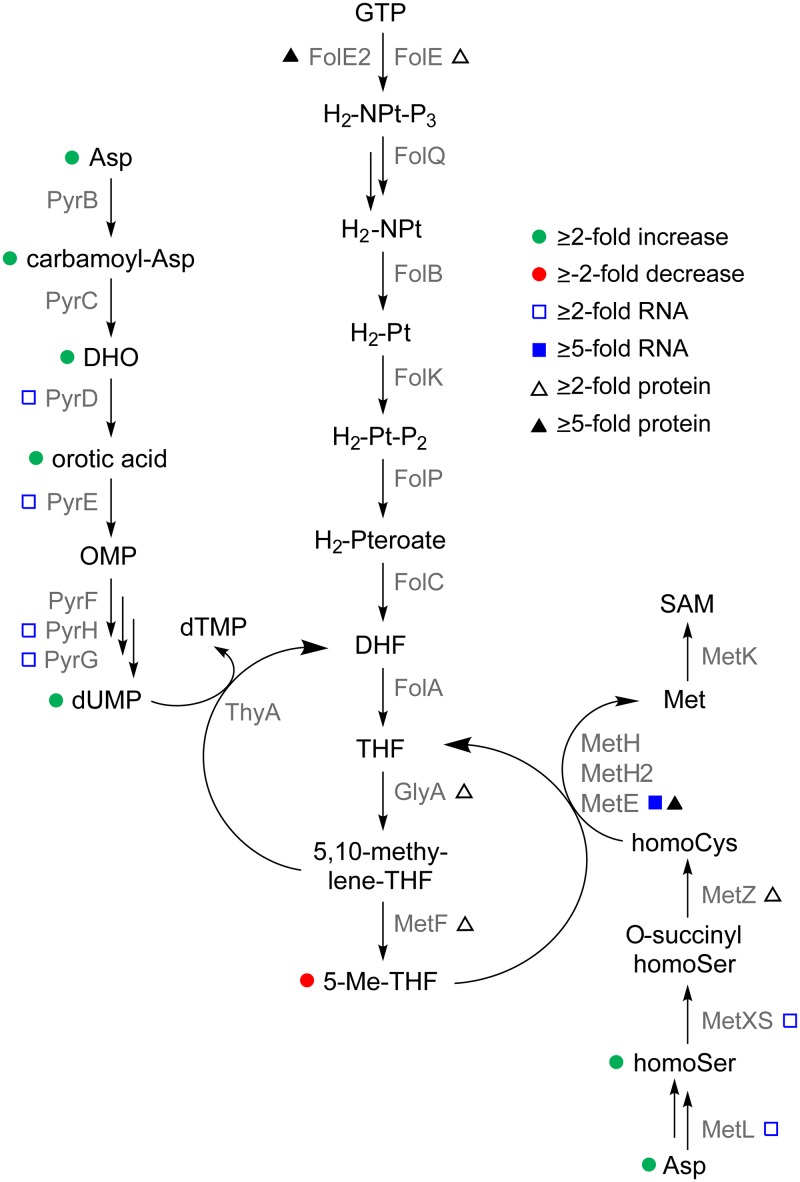
An omic readout of the effects of low-dose Tmp on one-carbon metabolism in B. thailandensis. The changes observed are indicated for the transcripts, proteins, and metabolites involved in the central one-carbon metabolic pathway. DHO, dihydroorotate; NPt, neopterin; Pt, pterin.

**FIG 7:**
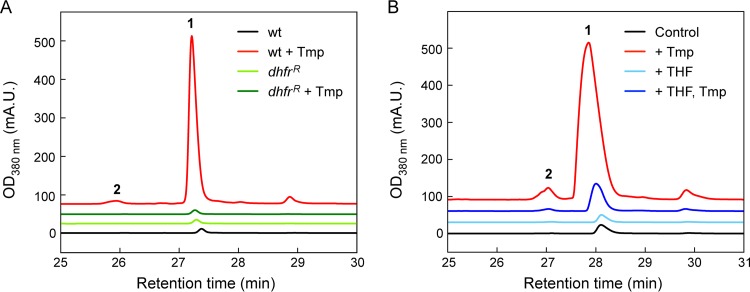
Low-dose Tmp induces malleilactone production and secondary metabolism by targeting DHFR. (A) Malleilactone production monitored by HPLC-MS in wt B. thailandensis cultures in control (black trace) or Tmp-treated (red trace) cultures, and in *dhfr*^r^-expressing B. thailandensis in control (light green) or Tmp-treated (dark green) cultures. (B) Malleilactone production monitored by HPLC-MS in B. thailandensis cultures that were DMSO treated (control, black trace) or supplemented with THF (light blue), with THF and Tmp (dark blue), or with Tmp only (red). Peaks corresponding to malleilactone A (1) and B (2) are labeled. THF was provided at a final concentration of 0.56 mM.

**(iii) Tmp targets DHFR to induce secondary metabolism but not via the stringent or SOS responses.** While the transcriptomic and proteomic data provided clues regarding the global effects of low-dose Tmp, they did not pinpoint a key gene or pathway that may explain the stimulatory response to the antibiotic. The primary metabolomic data, on the other hand, converged on one-carbon metabolism as a hot spot for various changes. Mapping the omics data onto this pathway showed that many genes/proteins were induced ≥2-fold (Fig. 6). We investigated this effect further by asking whether inhibition of the folate pathway by Tmp mediates the broad induction of secondary metabolism using high-performance liquid chromatography–mass spectrometry (HPLC-MS). Tmp failed to trigger secondary metabolite synthesis in a B. thailandensis mutant carrying a Tmp-resistant allele of *dhfr* at a neutral site (Fig. 7A and S3) (40). The levels of secondary metabolites that we did detect were commensurate with those in the DMSO control. Moreover, supplementation of Tmp-treated cultures with tetrahydrofolate (THF), the product of the folate pathway, almost completely abolished the stimulatory effect of the antibiotic (Fig. 7B). These data show that inhibition of DHFR by Tmp is necessary for the induction of *mal* and other secondary metabolite BGCs.

**FIG 8:**
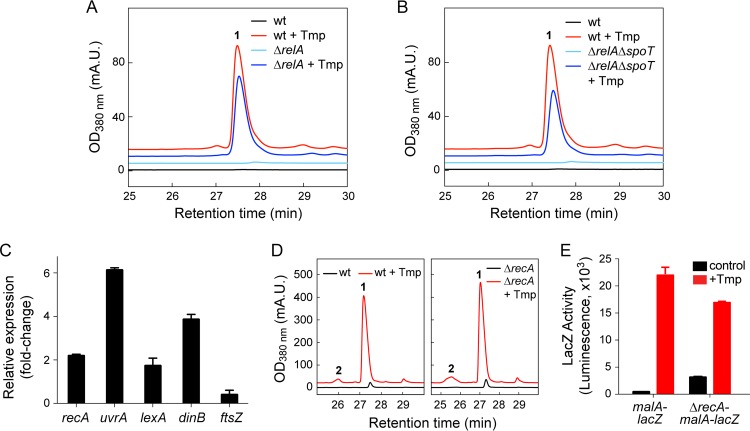
The stringent and SOS stress responses are not required for the stimulatory effect of Tmp. (A and B) Malleilactone synthesis measured by HPLC-MS in DMSO-treated (black trace) or Tmp-treated (red) cells for the wt control (A and B), Δ*relA* mutant (A), or Δ*relA* Δ*spoT* mutant (B). (C) Changes in the expression of SOS-related genes in response to Tmp determined by RT-qPCR. (D) Malleilactone levels measured by HPLC-MS in the absence (black trace) or presence (red) of Tmp in wt and Δ*recA* mutant B. thailandensis cultures. (E) Expression of the *mal* BGC in control (black bars) or Tmp-treated (red) B. thailandensis cultures with the genotypes indicated. The averages of three independent biological replicates are shown in panels C and E; error bars represent the SEM.

We reasoned that Tmp might induce *mal* by initiating a stress response caused by folate inhibition, the consequences of which include amino acid and thymidine starvation. We considered the stringent response (triggered by nutrient deprivation via RelA and SpoT) and the SOS response (triggered by DNA damage via RecA), as both are associated with Tmp treatment in E. coli (46–49). To test the involvement of the stringent response, we examined two insertional mutants, the Δ*relA* and Δ*relA* Δ*spoT* mutants (Tables S1 and S5) (48, 50), but found that Tmp activated secondary metabolism in both strains, though to a lesser degree in the double mutant (∼65%, Fig. 8A and B and S3). Moreover, treatment of B. thailandensis with various concentrations of Ser hydroxamate, a proven stringent response initiator, did not result in secondary metabolite biogenesis (Fig. S3). These results indicate that the stringent response is not sufficient for inducing *mal*.

The RNA-seq data showed that the SOS response was triggered by low-dose Tmp. We used reverse transcription-quantitative PCR (RT-qPCR) to verify these results and observed a 2- to 6-fold induction of *recA*, *lexA*, *uvrA*, and *dinB*, as well as 2.5-fold decreased *ftsZ* expression (Fig. 8C). Whether this response represents a homogenous weak SOS response to low-dose Tmp or a strong response by a heterogeneous subpopulation remains to be determined. We interrogated whether malleilactone production is under SOS control using an insertional *recA* mutant (Δ*recA* mutant) as well as a markerless *recA* deletion in the translational reporter background *malA-lacZ* (Δ*recA*-*malA-lacZ* mutant). Similar levels of malleilactone were elicited by Tmp in the wild type (wt) and Δ*recA* mutant, indicating that RecA, and by extension the SOS response, was not required (Fig. 8D). In agreement, the reporter constructs *malA-lacZ* and Δ*recA*-*malA-lacZ* gave similar levels of LacZ activity in the presence of Tmp (Fig. 8E). Together, these data show that low-dose Tmp elicits the SOS response in B. thailandensis, although it is not involved in triggering malleilactone biosynthesis. This conclusion is consonant with a prior report that secondary metabolism is not a component of the SOS regulon in this bacterium (51).

**FIG 9:**
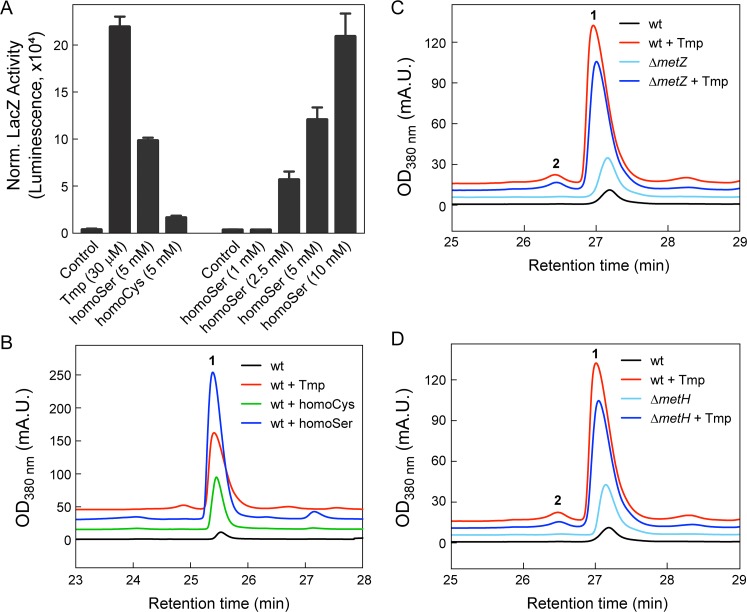
Homoserine induces expression of the *mal* BGC. (A) OD-normalized (Norm.) MalA-LacZ activity as a function of Tmp, homoSer, and homoCys relative to a DMSO control. (B) Malleilactone production monitored by HPLC-MS in wt B. thailandensis cultures treated with DMSO control (black trace), Tmp (30 μM, red), homoCys (5 mM, green), or homoSer (5 mM, blue). (C and D) Malleilactone synthesis measured by HPLC-MS in DMSO-treated control cultures (black trace) or in Tmp-treated (red) wt (C and D), Δ*metZ* mutant (C), and Δ*metH* mutant (D) cultures.

**(iv) Low-dose Tmp leads to enhanced homoSer and homoCys levels.** The results thus far allowed us to rule out global regulators and two stress response pathways as major factors affecting *mal* expression. Among the metabolic consequences of Tmp, we were intrigued by the distant effects on Met biosynthesis and the elevated levels of the immediate precursors homoSer and homoCys, which were increased ∼2-fold and ∼1.5-fold relative to a DMSO control, respectively. Given the increased concentrations of these precursors and that bacterial secondary metabolism can be regulated by *S*-adenosylmethionine (52), we investigated the effects of molecules upstream and downstream of Met (Fig. 3A). Remarkably, exogenous addition of homoSer and, to a lesser extent, homoCys, but not Cys, Met, and SAM, induced the *mal* BGC, as determined using a *malA-lacZ* reporter (Fig. 9A). A clear dose-dependent activation of *mal* was observed with homoSer, indicating that it is an inducer of the *mal* BGC (Fig. 9A). Further, HPLC-MS assays showed that both homoSer and homoCys triggered malleilactone biosynthesis (Fig. 9B). Homoserine not only elicited malleilactone but gave rise to a secondary metabolome that mirrored the response to Tmp, with the production of HMQs, thailandamide, and burkholdac (Fig. S4). Notably, the levels of malleilactone were greater with added homoSer than with Tmp, indicating that homoSer alone was able to recapitulate the response to low levels of the antibiotic (Fig. S4).

10.1128/mBio.03210-19.4 FIG S4 Homoserine addition mirrors the effects of Tmp on secondary metabolism. (A) Secondary metabolite production monitored by HPLC-MS in wt B. thailandensis cultures treated with DMSO control (black trace), homoCys (light blue), homoSer (dark blue), and Tmp (red). Shown is the elution profile at 280 nm, which captures a broader spectrum of metabolites produced by B. thailandensis. The peaks corresponding to malleilactone (1), HMQs (*a*), thailandamide (*b*), and burkholdac (*c*) are marked. Homoserine elicits a metabolome similar to that of Tmp, though the levels of 1 are elevated and those of *a*, *b*, and *c* are slightly lower. (B) Relative quantification of malleilactone production, determined by HPLC-MS, in wt B. thailandensis cultures treated with Tmp, homoCys, or homoSer at the final concentrations shown. The area under the malleilactone A peak was integrated and normalized for the OD_600_. Shown are the averages of three independent measurements; error bars represent the SEM. Download FIG S4, PDF file, 0.2 MB. Copyright © 2020 Li et al. 2020 Li et al. This content is distributed under the terms of the Creative Commons Attribution 4.0 International license.

We also examined mutants that are unable or hindered in their ability to convert homoSer to homoCys and homoCys to Met. A Δ*metZ* mutant strain which lacks *O*-succinyl-homoserine sulfhydrolase and should therefore accumulate homoSer produced slightly elevated levels of malleilactone compared to the wt in the absence of Tmp (Fig. 9C). Likewise, a Δ*metH* mutant strain, which lacks the B_12_-dependent Met synthase and should therefore accumulate homoCys and homoSer, also synthesized higher levels of malleilactone than did the wt (Fig. 9D). The addition of Tmp elevated the production of malleilactone in both strains. Taken together, these results indicate a link between low-dose Tmp, increased homoSer levels, and induction of malleilactone.

### MalR is required for homoSer-mediated induction of *mal*.

**FIG 10:**
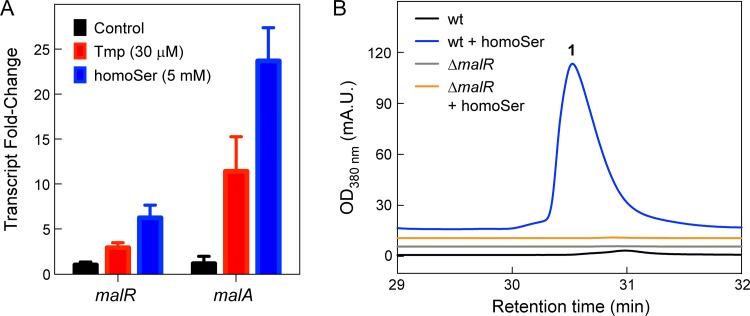
Homoserine induces *mal* through *malR*. (A) Induction of *malR* and *malA* by Tmp and homoSer, relative to a DMSO control, determined by RT-qPCR. Three independent biological replicates for both treated and untreated cells were used. Error bars represent the SEM. (B) Malleilactone production monitored by HPLC-MS in wt and Δ*malR* mutant B. thailandensis cultures in control (black and gray trace, respectively) or homoSer-treated cultures (blue and orange, respectively).

It was previously shown that MalR is required for the Tmp-mediated induction of *mal* (36). MalR is an orphan LuxR, a LuxR-type transcriptional regulator without a cognate LuxI, that is expressed divergently from *malA*. While the *lux* box to which MalR binds has been identified, its small-molecule ligand has not. We first showed that the addition of homoSer resulted in a 6- ± 1-fold induction of *malR* compared to a DMSO control using RT-qPCR (Fig. 10A), surpassing the effect of Tmp on *malR* expression. Next, using HPLC-MS, we could observe clear production of malleilactone in the wt in response to homoSer. The stimulatory response was completely abolished in a markerless *malR* deletion mutant (Fig. 10B). These data raise the following two possibilities: in one, homoSer induces a stress similar to that of Tmp that leads to secondary metabolite synthesis; alternatively, and perhaps more appealing, is a model in which Tmp treatment leads to enhanced homoSer levels, which in turn induce the *mal* BGC via *malR*. Whether homoSer serves as a ligand for MalR remains to be determined. This model is discussed further below.

### Conclusion.

We previously used high-throughput elicitor screening (HiTES) to identify Tmp as an inducer of malleilactone production in B. thailandensis (7). We subsequently expanded the use of HiTES to several other bacterial phyla to successfully elicit and characterize novel cryptic metabolites (53–57). In many cases, we found that low doses of toxins or antibiotics serve as the most effective elicitors of secondary metabolism; how these elicitors trigger cryptic metabolite production has not yet been examined (8, 54). In the current study, we have tackled the mechanism underlying this stimulatory response to better understand the regulation of silent gene clusters, focusing on Tmp and B. thailandensis.

**FIG 11:**
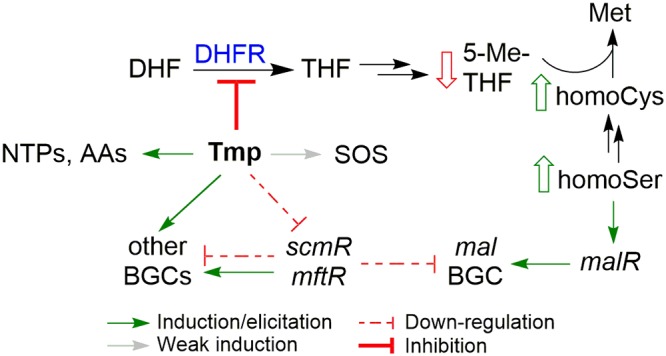
Summary of the effects of low-dose Tmp on B. thailandensis. Several pathways impinge on *mal*; the most important of these is inhibition of DHFR by Tmp leading to long-range inhibition of Met biosynthesis, as a result of lowered levels of 5-Me-THF, and to increased levels of homoCys and homoSer. The latter induces *mal* via *malR*. Low-dose Tmp treatment also triggers the SOS response, leads to enhanced NTPs and amino acids, and downregulates *scmR* and *mftR*, resulting in modulation of numerous BGCs in B. thailandensis, though these do not constitute the major pathway by which Tmp induces *mal*.

We find that low-dose Tmp targets DHFR to induce secondary metabolite synthesis globally, but its stimulatory effects do not depend on the stringent or SOS stress response. The general features observed with low-dose Tmp have much in common with those observed in other bacteria, typically assayed at lethal concentrations and shorter time points. A key element in our studies is the accumulation of homoSer and, weakly, homoCys, which is sufficient for induction of the *mal* BGC. To our knowledge, these metabolites have previously not been identified as elicitors of cryptic metabolites in any bacterium. We propose that Tmp at least partially activates secondary metabolism by depleting the reduced folate pools, thereby putting a stranglehold on one-carbon anabolic reactions. A direct consequence is impedition of homocysteine methylation, leading to accumulated homoSer, which in turn induces *malR* to activate the *mal* BGC (Fig. 11). Interestingly, Met has been proposed as a precursor to malleilactone, with the C-2, C-3, and C-4 carbons of Met being incorporated into the propionyl group of malleilactone (17). Because the interconversion of homoCys and Met is reversible, it is possible that homoCys or homoSer serves to induce the *mal* BGC and as a substrate for the synthesis of the virulence factor. Regardless of whether these intermediates serve as a substrate, our results reveal a metabolite-centric mechanism for the induction of the *mal* BGC in response to low-dose Tmp.

We do not yet know how B. thailandensis senses homoSer and/or homoCys to couple their build-up to the induction of secondary metabolism. Given the special sensitivity of malleilactone production to folate pathway inhibition, it is possible that homoSer and/or homoCys can serve as a small-molecule ligand, a so-called coinducer, to allosterically activate MalR, the pathway-specific regulator of the *mal* BGC. Speculatively, why might it make sense for B. thailandensis to associate increases in Met precursors with secondary metabolite activation? Sensing precursors may be an indirect, but potentially more sensitive, way of gauging the levels of end products of metabolic pathways (58). If impaired Met biosynthesis is due to a nearby competitor, these intermediates could serve as early signals to activate secondary metabolite production, possibly as a defense mechanism (59).

We conclude that multiple inputs impinge on the production of malleilactone. Chief among these are the long-range effects on Met biosynthesis which lead to an accumulation of homoSer, an effective elicitor of the *mal* cluster. However, there are additional impacts of low-dose Tmp treatment, as outlined above. While bacteria respond to lethal concentrations of Tmp with a clear stringent phenotype, the response to low-dose Tmp is mixed. It mirrors the stringent response with respect to amino acid biosynthesis. However, the accumulation of NTPs suggests a nutrient surplus, perhaps indicative of the hormetic effects of Tmp, in which inhibitory concentrations shut down primary metabolism but subinhibitory doses lead to a metabolically more active state. The induction of many large biosynthetic machines is consistent with B. thailandensis paradoxically interpreting low-dose Tmp treatment as a form of nutrient excess. Future studies will further explore the molecular connection between low-dose Tmp and this apparent nutrient excess. They will also delve into the mechanisms by which Tmp, and other antibiotics identified so far, induce secondary metabolite BGCs beyond *mal* in B. thailandensis.

## MATERIALS AND METHODS

### Bacterial strains and growth conditions.

B. thailandensis E264 was used throughout this study. It was routinely cultured in LB-morpholinepropane sulfonic acid (LB-MOPS), which consists of LB (Becton, Dickinson) supplemented with 50 mM MOPS (Fisher), with the pH adjusted to 7.0 using NaOH. All antibiotics were obtained from Sigma. Trimethoprim was dissolved in DMSO and used at a final concentration of 30 μM. Table S1 lists all bacterial strains, gene deletion mutants, insertional mutants, transposon (Tn) mutants, and reporter strains constructed or used in this study, and Table S5 lists all primers used. The OD_600_ was measured using a Cary UV-Vis spectrophotometer.

### LacZ reporter assays.

Wild-type B. thailandensis (or the desired mutant) was streaked out onto an LB-agar plate from frozen cell stocks and the plate incubated at 30°C overnight. The resulting colonies were used to inoculate 5 ml of LB-MOPS medium in a 14-ml bacterial culture tube, which was incubated at 30°C and 200 rpm overnight. The culture was used to inoculate a 125-ml Erlenmeyer flask (containing 20 ml LB-MOPS) to an initial OD_600_ of 0.05. When necessary, Tmp was added to a final concentration of 30 μM. A DMSO control culture was always included as well, to which only the same volume of DMSO was added. The concentrations of other additives (i.e., amino acids) are indicated in the figure legends (Fig. 7, 9, and 10). The cultures were then grown at 30°C and 200 rpm for 12 h. Then, 65 μl was transferred to a white-bottom 96-well plate, supplemented with 35 μl of Beta-Glo reagent (Promega), which was prepared according to manufacturer’s instructions, followed by a 2:1 dilution with water. The mixture was incubated in the dark at room temperature (RT) for 1 h with occasional agitation before measuring endpoint luminescence on a Synergy H1 microplate reader. Luminescence counts were normalized for OD_600_. Experiments were typically carried out in three biological replicates for the control (DMSO) and Tmp/amino acid-treated samples.

### Detection of malleilactone by HPLC-MS.

Low-resolution HPLC-MS analysis was performed on a 1260 Infinity series HPLC system (Agilent) equipped with an automated liquid sampler, a diode array detector, and a 6120 series electrospray ionization (ESI) mass spectrometer using an analytical Luna C_18_ column (5 μm, 4.6 by 100 mm; Phenomenex) operating at 0.5 ml/min. Compounds were resolved with an isocratic step, with 25% acetonitrile (MeCN) in H_2_O over 5 min, followed by a gradient of 25 to 100% MeCN over 27 min. Both MeCN and H_2_O contained 0.1% (vol/vol) formic acid (FA). Flask cultures (20 ml in a 125 ml-Erlenmeyer flask) were prepared in an identical fashion as described above for the luminescence assays. For the detection of malleilactone, the cultures were grown for 24 h (OD_600_, ∼5.0) at 30°C and 200 rpm. Each culture was then extracted with 20 ml of ethyl acetate. The organic layer was dried *in vacuo* and dissolved in 400 to 1,000 μl methanol (MeOH), which was filtered and analyzed by HPLC-MS. Malleilactone production was monitored at 380 nm. The data were normalized for OD_600_.

### RNA-seq.

Overnight cultures of wt B. thailandensis were prepared as described above and used to inoculate six 125-ml Erlenmeyer flasks, each containing 20 ml LB-MOPS, at an initial OD_600_ of 0.05. Three cultures contained 30 μM Tmp, and the other three contained DMSO (control). The cultures were grown to an OD_600_ of ∼1.0, at which point ∼5 × 10^8^ cells were isolated from each replicate by centrifugation. Total RNA was isolated using the RNeasy minikit (Qiagen), and its integrity was verified by application onto an Agilent Bioanalyzer. Twenty micrograms of RNA for each replicate was submitted to the Genomics Core Facility at Princeton University for library construction and sequencing. rRNA was depleted using the Ribo-Zero rRNA removal kit (Illumina). Sequencing libraries were constructed using the TruSeq stranded total RNA library prep kit (Illumina). Sequencing was performed with the NovaSeq SP 100-nucleotide (nt) flow cell. A total of 37 million reads, consisting of 2 × 50-nt paired-end reads, were obtained for each replicate.

Quality control of the sequencing data was carried out using FastQC. Raw reads were mapped back to the genome of B. thailandensis E264 using Bowtie2, and read counts were determined by featureCount. Differential gene expression analysis was conducted with DESeq2.

### TMTc+ proteomics.

Six B. thailandensis cultures were initiated with or without Tmp as described above in 125-ml Erlenmeyer flasks (each containing 20 ml) and grown to an OD_600_ of ∼1.0. Then, 1 ml was harvested from each by centrifugation (10,000 × *g* for 1 min) in an Eppendorf tube, washed 3 times with 1 ml ice-cold phosphate-buffered saline (PBS), and then stored at –80°C. Samples were generated in an identical fashion at an OD_600_ of ∼5.0 (Tmp treated) and compared to a DMSO control (OD_600_, ∼6.5).

TMT-labeled peptides were prepared mostly as previously described (60, 61). Cell pellets were resuspended in 600 μl of 6 M guanidine hydrochloride (GnHCl), 2% cetyltrimethylammonium bromide (CTAB), 50 mM HEPES, 1 mM EDTA, and 5 mM dithiothreitol (DTT) (pH 7.4). Cells were vortexed to homogeneity, divided into 200-μl aliquots, with two per growth condition, and placed on ice. They were then lysed with a probe sonicator set to 50% power for 2 × 25 s. Proteins were denatured further at 60°C for 20 min. After cooling, cysteines were alkylated by the addition of 20 min mM *N*-ethylmaleimide for 20 min, followed by quenching with DTT (10 mM).

The protein solutions (200 μl) were charged with 800 μl MeOH, vortexed for 1 min, supplemented with 400 μl chloroform, vortexed for 1 min, followed by addition of 600 μl water and vortexing (1 min). The precipitated proteins were brought to the extraction interface by centrifugation (2 min, 20,800 × *g*), followed by removal of the upper layer. The protein interface was washed and pelleted from the chloroform phase by the addition of 600 μl MeOH, followed by vortexing (1 min) and centrifugation as described above. The wash solution was removed, and the pellet was washed with 1 ml MeOH. After the removal of MeOH, the pellets were resuspended in 100 μl of 6 M GnHCl and 10 mM EPPS (3-[4-(2-hydroxyethyl)-1-piperazinyl]propane sulfonic acid) (pH 8.5). After mixing at ∼1,000 rpm for 30 min, 15 μl was removed for digestion.

The resuspended proteins were digested by adding 75 μl of LysC at 26.7 ng/μl in 10 mM EPPS (pH 8.5), followed by vortexing (10 s) and incubation at RT overnight. Further proteolysis was performed by the addition of trypsin and LysC (60). The peptides were dried in a SpeedVac and resuspended in 30 μl of 200 mM EPPS (pH 8.0) to a concentration of 0.5 g/liter. Ten microliters were removed from each resuspension and charged with 2 μl of different TMT-isobaric mass tag *N*-hydroxysuccinimide (NHS) ester (20 g/liter). The acylations were reacted at RT and 1,000 rpm and quenched with 0.5 μl of 5% hydroxylamine for 20 min, followed by 1 μl of 5% phosphoric acid.

Peptides were enriched from the acidified TMT labeling reactions by solid-phase extraction using a Waters Sep-Pak μElution 96-well plate (3 mg/well). One well per multiplexed quantitative proteomics experiment was wetted with 200 μl MeOH and then hydrated with 400 μl water. Labeling reaction mixtures were pooled and diluted into 200 μl and allowed to adsorb onto the C_18_ silica column under gravity flow. The adsorbed peptides were washed with 100 μl water, followed by centrifugation for 1 min at 180 rpm. The peptides were eluted with sequential additions of 100 μl of 35% acetonitrile (+ 1% formic acid [FA]) and 100 μl of 70% acetonitrile (+1% FA). Eluates were pooled and dried in a SpeedVac. The peptides were resuspended in 200 μl of 1% FA and subjected to quantitative multiplexed proteomics by nano-ultraperformance liquid chromatography-tandem mass spectrometry (nanoUPLC-MS/MS).

TMTc+ analysis was performed as previously described (41). The instrument was operated in data-dependent mode with a survey scan performed at a resolution setting of 120k (*m/z* 200) with a scan range of *m/z* 500 to 1,400, an RF (radio frequency) lens of 60%, automatic gain control (AGC) target of 10^6^, and a maximum injection time of 100 ms. Only charge states of 2+ were included. A dynamic exclusion window of 60 s with a mass tolerance of ±10 ppm was used. Peptides with a minimum intensity of 3 × 10^6^ or higher were subjected to an MS^2^ scan using an isolation window of 0.4 using the quadrupole. Peptides were fragmented with a high-energy collisional dissociation (HCD) collision energy of 32%, and a mass spectrum was acquired using the Orbitrap analyzer with a resolution of 30k, an AGC target of 5 × 10^4^, and a maximum injection time of 120 ms. Data analysis to obtain the fold change for each protein was carried out with BACIQ (62).

### Targeted and unbiased analysis of omics results.

For targeted analysis, the B. thailandensis proteome was first annotated with the eggNOG-mapper (http://eggnogdb.embl.de/#/app/emapper). Significantly regulated gene lists were extracted from transcriptomics and proteomics data as mentioned above. The fraction of proteins within a category was then calculated. For unbiased analysis, RNA and protein expression levels were first normalized and then combined for K-means clustering via the “kmeans” function in R. Lists for different clusters were then submitted to the DAVID website (https://david-d.ncifcrf.gov/) for functional annotation and enrichment analysis. Significantly enriched terms were plotted via R.

### Global primary metabolite analysis.

Overnight cultures of B. thailandensis were prepared as described above and diluted to an initial OD_600_ of 0.05 in 125-ml Erlenmeyer flasks containing 20 ml of LB-MOPS. Three flasks were supplemented with a final concentration of 30 μM Tmp, and the other three were supplemented with DMSO. The cultures were grown at 30°C and 200 rpm to an OD_600_ of ∼3.5 (Tmp treated) and 4.5 (DMSO control). Primary metabolites were isolated by filtering ∼300 μl of each culture onto a 50-mm nylon membrane filter and immediately quenching the membrane in extraction solvent (2:2:1 MeCN-MeOH-water with 0.1 M formic acid) that had been precooled at –20°C. The extraction process continued for 15 min. Then, each extract was neutralized by adding 300 μl of 15% (wt/vol) NH_4_HCO_3_ and centrifuged at 12,000 × *g* for 10 min at 4°C. The supernatants were analyzed by reversed-phase ion-pairing HPLC coupled to a high-resolution, high-accuracy electrospray ionization (ESI) mass spectrometer (Thermo Q Exactive mass spectrometer), which was operated in negative ion detection in full scan mode, as described previously (63). Primary metabolites were verified by high-resolution mass and retention time matches to authentic standards. Metabolites were quantified in the MAVEN software using integrated peak areas from extracted ion chromatograms (64). Metabolite levels were expressed as the relative fold change to the DMSO-treated samples. Three biological replicates were averaged before fold changes were calculated.

### Creation of site-specific deletion mutants.

All deletion mutants, with the exception of the markerless *malR* mutant, were constructed by natural competence transformation with linear DNA fragments (65), which were created by joining three fragments corresponding to upstream and downstream 1-kb regions flanking the gene to be deleted and an antibiotic resistance gene (kanamycin resistance [Kan^r^] or tetracycline resistance [Tet^r^]). Competent B. thailandensis cells were generated by growing the bacteria overnight in 5 ml low-salt LB medium at 37°C and 200 rpm. The overnight culture was used to inoculate 3 ml of M63 medium in a 14-ml sterile culture tube grown at 37°C and 200 rpm. After ∼10 h, the cells were pelleted by centrifugation at 12,000 × *g* for 2 min, and the supernatant was discarded. The cells were resuspended in 100 μl of M63 medium, and 50 μl of the suspension was transferred into a sterile 1.5-ml Eppendorf tube. Approximately 500 ng of linear DNA fragment was mixed with the cells by gently tapping the tube, and the resulting mixture was incubated at RT for 30 min. The mixture was then transferred into 2 to 3 ml of M63 medium in a 14-ml culture tube and grown at 37°C and 200 rpm overnight. The overnight culture was pelleted by centrifugation, and the cells were resuspended in ∼80 μl of M63 medium and plated on low-salt LB agar supplemented with the appropriate antibiotic(s). The plate was then incubated at 37°C for 24 h or until colonies developed. The mutant colonies were verified by PCR and sequencing. The *malR* markerless deletion mutant was created as previously described in detail for *scmR* (37). The primers used to generate the *malR* deletion are listed in Table S5.

### RT-qPCR analysis.

Flask cultures of B. thailandensis were prepared as described above. The cultures were then grown at 30°C and 200 rpm for 12 h. The OD_600_ for each culture was determined, 100 μl of each was dispensed into a 1.5-ml RNase-free Eppendorf tube, and the cells were pelleted by centrifugation at 12,000 × *g* for 3 min. The supernatant was discarded, and the cells were immediately frozen in liquid N_2_ for 5 min. The samples were then stored at –80°C. RNA isolation was carried out using the Qiagen RNeasy kit. RNA integrity was confirmed by gel electrophoresis. Contaminating DNA was removed from the sample using the DNA-free kit. Finally, the RNA was converted into cDNA using the iScript kit (Life Technologies) with random hexamers as primers and 500 ng of each RNA sample as the template.

RT-qPCR primers were designed manually to give an amplicon length of 100 to 130 bp and a melting temperature of 60°C. The genomic DNA (gDNA) of B. thailandensis was isolated and used as the template to obtain each amplicon, which was then purified and quantified. A series of standards were generated for each amplicon ranging from ∼2 pg/μl down to ∼200 attograms (ag)/μl in four successive 10-fold dilution steps and used in quantification (see below). qPCR analysis was performed on a CFX96 real-time PCR detection system (Bio-Rad). The reaction was carried out in hard-shell, clear 96-well qPCR plates and utilized the iTaq Universal SYBR green Supermix (Bio-Rad). Each well contained 8 μl of iTaq Supermix, 1 μl of standard DNA or cDNA, 1 μl of each primer, and 5 μl of nuclease-free water. The PCR cycle consisted of a 1-min incubation (95°C), followed by 42 cycles of a 2-step amplification protocol (5 s at 95°C and then 30 s at 59.5°C). This was followed by a denaturation cycle to determine the specificity of the reaction, where a single species was observed for all experiments reported.

To determine the levels of each transcript, the quantification cycle (*C_q_*) numbers were determined in triplicate and then converted to amplicon concentration using linear fitting. The resulting value was then normalized for total cell numbers determined in the RNA isolation step and further normalized to the DMSO control sample to give the fold change for that amplicon.
