# Supplementary figures and images for: Multi-Omic Analyses Provide Links between Low-Dose Antibiotic Treatment and Induction of Secondary Metabolism in Burkholderia thailandensis
Source: mBio. 2020 Feb 25;11(1):e03210-19. doi: 10.1128/mBio.03210-19 (PMC7042699; doi:10.1128/mBio.03210-19)

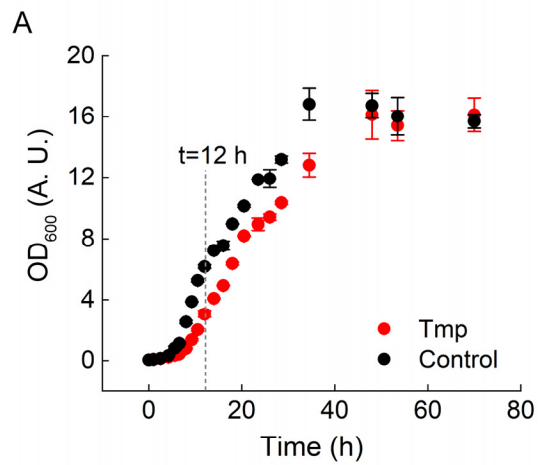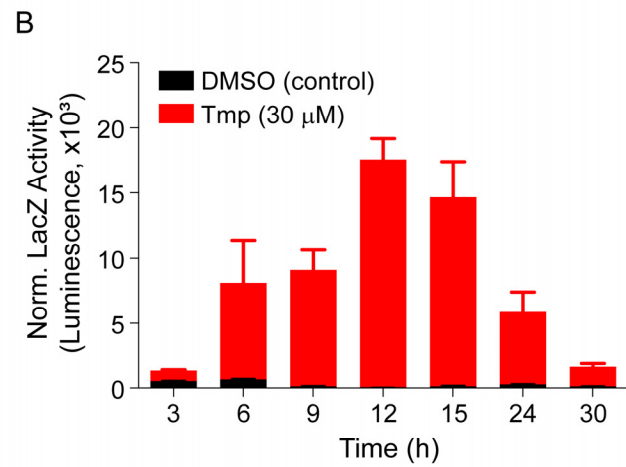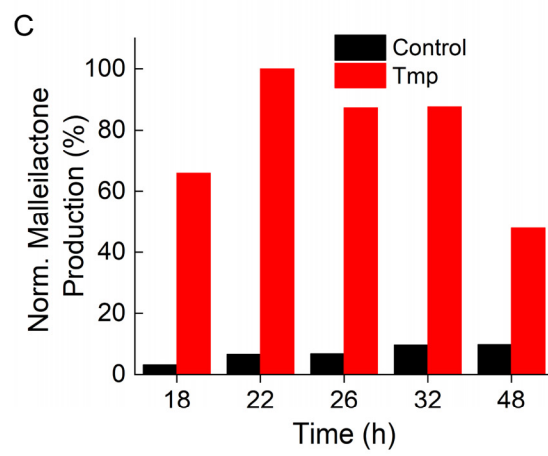

Supplement: FIG S1 [file mBio.03210-19-sf001.pdf]

A

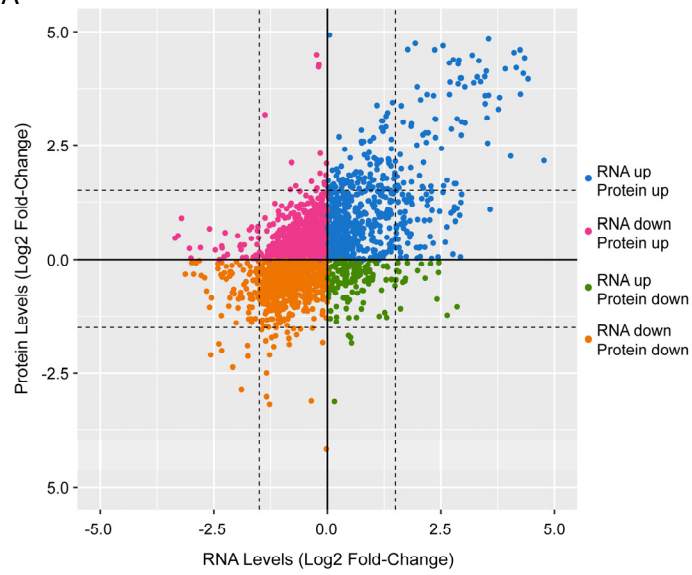

B

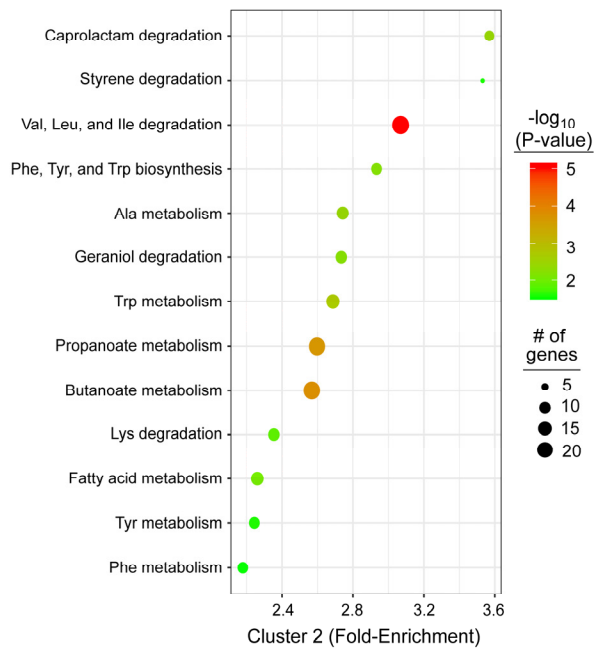

C

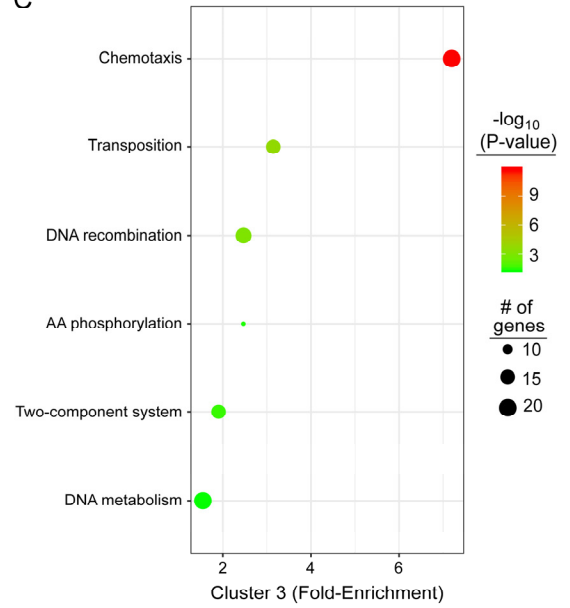

D

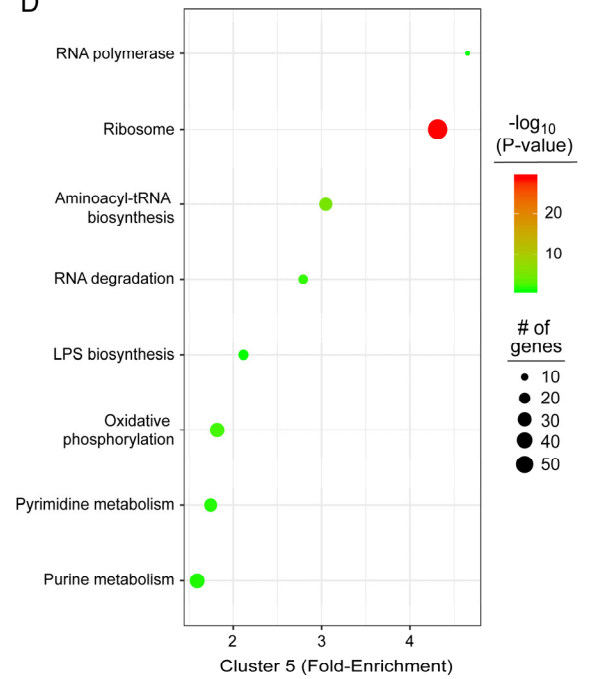

Supplement: FIG S2 [file mBio.03210-19-sf002.pdf]

A

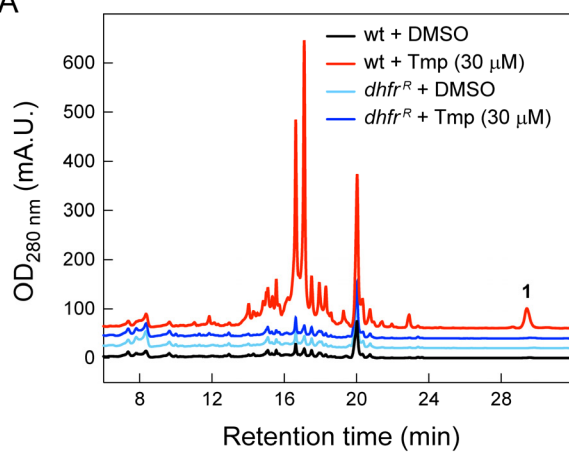

C

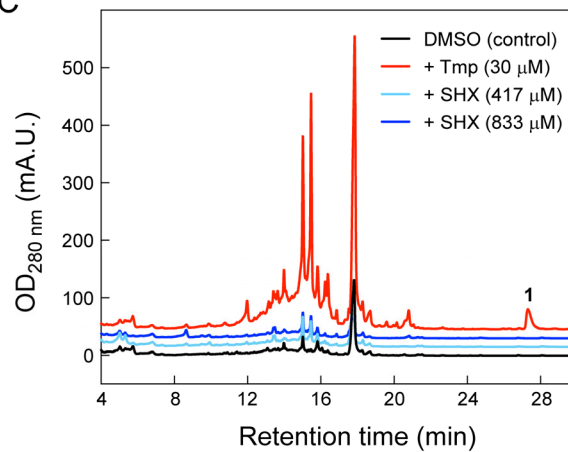

B

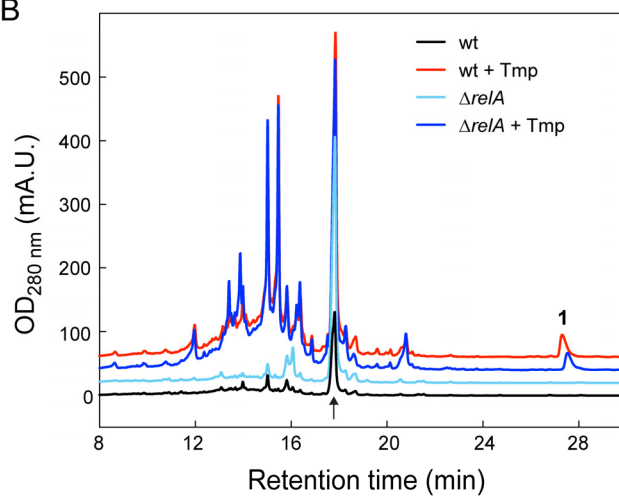

Supplement: FIG S3 [file mBio.03210-19-sf003.pdf]

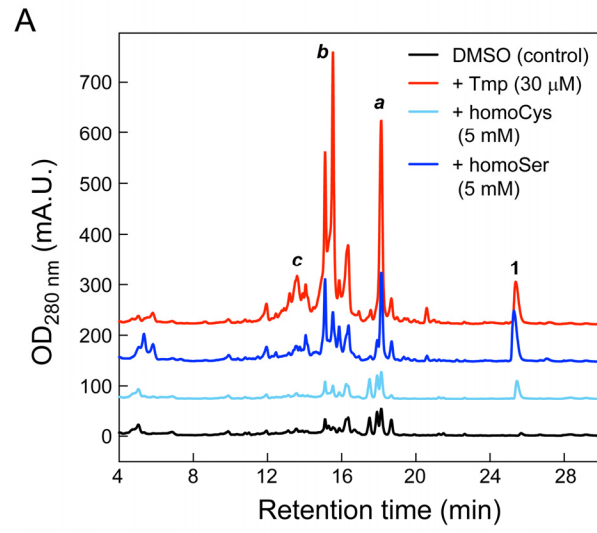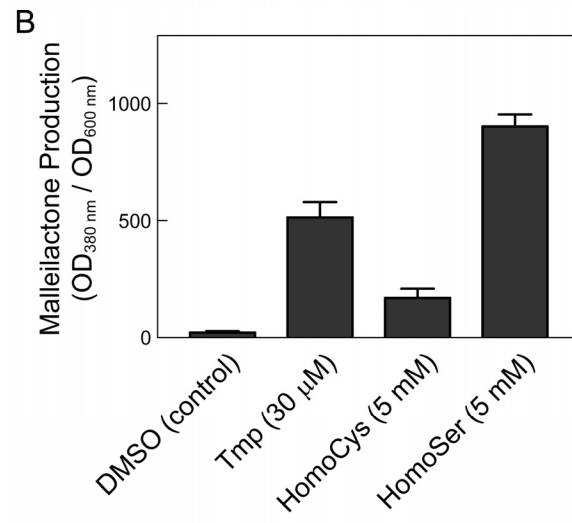

Supplement: FIG S4 [file mBio.03210-19-sf004.pdf]
