# Supplementary material for: Multi-Omic Analyses Provide Links between Low-Dose Antibiotic Treatment and Induction of Secondary Metabolism in Burkholderia thailandensis
Source: mBio. 2020 Feb 25;11(1):e03210-19. doi: 10.1128/mBio.03210-19 (PMC7042699; doi:10.1128/mBio.03210-19)
Supplement: TABLE S1 [file mBio.03210-19-st001.docx]

**Table S1** Bacterial strains and plasmids generated or used in this study.

| **Bacteria strains** | **Description** | **Source** |
| --- | --- | --- |
| ***E. coli*** |  |  |
| DH5α | F- Φ80*lacZ* Δ *M15* Δ *(lacZYA-argF)U169 hsdR17*(rK - mK +) *recA1 endA1 phoA supE44 thi-1* *gyrA96 relA1 λ-* | NEB |
| JV36 | *E. coli* conjugation donor | (1) |
|  |  |  |
| ***B. thailandensis*** |  |  |
| E264 | Wild type | ATCC |
| ML#44 | *malA*::T8*-lacZ*, malleilactone BGC reporter strain | (2) |
| AL18 | *ΔmetZ* insertional mutant of E264, tet^R^ | This study |
| AL19 | *ΔmetH* insertional mutant of E264, tet^R^ | This study |
| AL29 | *ΔmalR* markerless mutant of E264 | This study |
| DM8 | *ΔrelAΔspoT* insertional mutant of E264, kan^R^, tet^R^ | This study |
| DM9 | *ΔrecA* insertional mutant of E264, tet^R^ | This study |
| DM10 | *ΔrecA* markerless mutant of ML#44 | This study |
| DM1  AL25 | *ΔscmR* markerless mutant of E264  *ΔmftR* insertional mutant of E264, kan^R^ | (3)  This study |
|  |  |  |
| **Plasmids** |  |  |
| pEX18Kan-PheS | Conjugation vector and source of Kan^R^ gene, Kan^R^ | (4) |
| pEX18Tet-PheS | Source of Tet^R^ gene | (4) |
|  |  |  |
|  |  |  |

1. Blodgett JA, Oh DC, Cao S, Currie CR, Kolter R, Clardy, J. 2010. Common biosynthetic origins for polycyclic tetramate macrolactams from phylogenetically diverse bacteria. Proc Natl Acad Sci USA 107:11692–11697.
2. Gallagher LA, Ramage E, Patrapuvich R, Weiss E, Brittnacher M, Manoil C. 2013. Sequence-defined transposon mutant library of *Burkholderia thailandensis*. MBio 4:e00604-13.
3. Mao D, Bushin LB, Moon K, Wu Y, Seyedsayamdost MR. 2017. Discovery of *scmR* as a global regulator of secondary metabolism and virulence in *Burkholderia thailandensis* E264. Proc Natl Acad Sci USA 114:E2920–E2928.
4. Barrett AR, Kang Y, Inamasu KS, Son MS, Vukovich JM. Hoang TT. 2008. Genetic tools for allelic replacement in *Burkholderia* species. Appl Environ Microbiol 74:4498-4508.
