# Supplementary material for: Multi-Omic Analyses Provide Links between Low-Dose Antibiotic Treatment and Induction of Secondary Metabolism in Burkholderia thailandensis
Source: mBio. 2020 Feb 25;11(1):e03210-19. doi: 10.1128/mBio.03210-19 (PMC7042699; doi:10.1128/mBio.03210-19)
Supplement: TABLE S2 [file mBio.03210-19-st002.docx]

**Table S2** All genes up-regulated (top) or down-regulated (bottom) ≥5-fold in response to Tmp as determined by RNA-Seq at OD_600_ ~1.0. The averages of three independent measurements are shown. Standard errors were typically <10% of the mean values reported.

| **Locus tag** | **Fold change** | **Joint Genome Institute product name** | |
| --- | --- | --- | --- |
| BTH_I1921 | 81.2 | gp26 | |
| BTH_II2113 | 51.0 | hypothetical protein | |
| BTH_II2114 | 38.4 | putative 4-oxalocrotonate tautomerase | |
| BTH_I3247 | 27.2 | aldehyde dehydrogenase family protein | |
| BTH_II2236 | 26.4 | porin | |
| BTH_II2095 | 21.4 | diaminopimelate decarboxylase, putative | |
| BTH_I0617 | 20.8 | hypothetical protein | |
| BTH_II2098 | 20.3 | malonyl CoA-acyl carrier protein transacylase | |
| BTH_II2099 | 19.9 | AMP-binding domain protein | |
| BTH_II2097 | 19.4 | lipoprotein, putative | |
| BTH_II2090 | 19.3 | syringomycin synthesis regulator SyrP, putative | |
| BTH_II2091 | 19.0 | adenosylmethionine-8-amino-7-oxononanoate aminotransferase, putative | |
| BTH_II2096 | 18.9 | long-chain-fatty-acid--CoA ligase, putative | |
| BTH_II2093 | 17.9 | polyketide synthase, putative | |
| BTH_I1606 | 17.3 | methionine synthase (B12-independent) (EC 2.1.1.14) | |
| BTH_II2089 | 16.7 | hypothetical protein | |
| BTH_II0207 | 16.4 | hypothetical protein | |
| BTH_II2092 | 15.1 | aldehyde dehydrogenase family protein | |
| BTH_II2238 | 14.8 | OpgC protein, putative | |
| BTH_II0206 | 13.9 | hypothetical protein | |
| BTH_II1282 | 13.7 | serine/threonine kinase | |
| BTH_II0204 | 13.6 | peptide synthetase, putative | |
| BTH_II2237 | 12.8 | MmgE/PrpD family protein | |
| BTH_I2846 | 12.6 | lipoprotein, putative | |
| BTH_II2269 | 12.4 | hypothetical protein | |
| BTH_II0031 | 12.3 | hypothetical protein | |
| BTH_II0033 | 12.0 | LysE family protein | |
| BTH_I2945 | 11.9 | peptidase, M1 family | |
| BTH_II0025 | 11.8 | hypothetical protein | |
| BTH_II1283 | 11.8 | hypothetical protein | |
| BTH_I2361 | 11.7 | phosphotransferase enzyme family protein, putative | |
| BTH_II0032 | 11.7 | hypothetical protein | |
| BTH_II2094 | 11.7 | ketol-acid reductoisomerase (EC 1.1.1.86) | |
| BTH_II0205 | 11.5 | hypothetical protein | |
| BTH_I2363 | 11.4 | polyketide synthase | |
| BTH_II0166 | 11.4 | flagellar biosynthetic protein FliQ, putative | |
| BTH_II2088 | 11.3 | thiotemplate mechanism natural product synthetase | |
| BTH_II2130 | 11.1 | transcriptional regulator, LysR family | |
| BTH_I2364 | 11.1 | peptide synthetase, putative | |
| BTH_I2359 | 11.1 | pyridine nucleotide-disulphide oxidoreductase, class II | |
| BTH_I2362 | 11.1 | acyl-CoA dehydrogenase domain protein | |
| BTH_II1125 | 10.6 | hypothetical protein | |
| BTH_II2281 | 10.5 | translation initiation inhibitor | |
| BTH_II0173 | 10.5 | Flagellar assembly protein FliH | |
| BTH_I2360 | 10.4 | nonribosomal peptide synthetase, putative | |
| BTH_I2365 | 10.1 | polyketide synthase | |
| BTH_II1385 | 10.0 | YceI like family protein | |
| BTH_I1920 | 9.9 | gp24 | |
| BTH_II0029 | 9.9 | hypothetical protein | |
| BTH_II2106 | 9.8 | efflux transporter, RND family, MFP subunit | |
| BTH_II2129 | 9.8 | major facilitator family transporter | |
| BTH_II2300 | 9.7 | hypothetical protein | |
| BTH_I3280 | 9.5 | transcriptional regulator, LysR family | |
| BTH_I2366 | 9.3 | polyketide synthase | |
| BTH_II2271 | 9.2 | hypothetical protein | |
| BTH_II2105 | 9.1 | hydrophobe/amphiphile efflux family protein | |
| BTH_II0692 | 8.9 | hypothetical protein | |
| BTH_II0691 | 8.9 | acyltransferase family protein | |
| BTH_II0764 | 8.9 | hypothetical protein | |
| BTH_II1387 | 8.5 | RNA polymerase sigma-70 factor, ECF subfamily | |
| BTH_II0172 | 8.3 | flagellar motor switch protein FliG, putative | |
| BTH_II0165 | 8.3 | flagellar biosynthetic protein FliR, putative | |
| BTH_I2358 | 8.2 | lipase/esterase | |
| BTH_II2112 | 8.2 | lipoprotein, putative | |
| BTH_II0279 | 8.2 | methoxy mycolic acid synthase 2 | |
| BTH_I0445 | 8.1 | DNA polymerase III, alpha subunit (EC 2.7.7.7) | |
| BTH_I0303 | 8.1 | related to SH3-domain protein Cyk3 | |
| BTH_II2071 | 7.9 | malate/L-lactate dehydrogenase family protein | |
| BTH_II2267 | 7.9 | pilus assembly protein | |
| BTH_II2265 | 7.9 | peptidase, putative | |
| BTH_II0072 | 7.8 | hemolysin activator protein, HlyB family, putative | |
| BTH_II1219 | 7.8 | phosphopantetheine attachment site domain protein | |
| BTH_II0174 | 7.8 | flagellum-specific ATP synthase FliI | |
| BTH_I0078 | 7.8 | hypothetical protein | |
| BTH_II2064 | 7.8 | oxidoreductase, putative | |
| BTH_II0028 | 7.8 | hypothetical protein | |
| BTH_II0003 | 7.8 | hypothetical protein | |
| BTH_II0168 | 7.7 | flagellar motor switch protein FliN | |
| BTH_II1663 | 7.7 | pyruvate ferredoxin/flavodoxin oxidoreductase family protein | |
| BTH_II2104 | 7.7 | RND efflux system, outer membrane lipoprotein, NodT family | |
| BTH_II0548 | 7.7 | glycosyl transferase, group 2 family protein | |
| BTH_II0281 | 7.7 | JamB | |
| BTH_II0547 | 7.7 | exopolysaccharide tyrosine-protein kinase, putative | |
| BTH_II0102 | 7.7 | hypothetical protein | |
| BTH_II0544 | 7.6 | UDP-glucose 6-dehydrogenase | |
| BTH_II1772 | 7.6 | HD domain protein | |
| BTH_II0178 | 7.6 | hypothetical protein | |
| BTH_II2107 | 7.6 | lipH | |
| BTH_I1262 | 7.5 | hypothetical protein | |
| BTH_II1218 | 7.5 | AMP-binding domain protein | |
| BTH_II0253 | 7.5 | ompA family protein | |
| BTH_II0278 | 7.5 | JamB | |
| BTH_I1263 | 7.5 | hypothetical protein | |
| BTH_II1372 | 7.5 | hypothetical protein | |
| BTH_II2363 | 7.4 | GGDEF domain protein | |
| BTH_II1711 | 7.4 | hypothetical protein | |
| BTH_II1664 | 7.4 | polyketide synthase, putative | |
| BTH_II0237 | 7.4 | pdhA | |
| BTH_II0169 | 7.4 | hypothetical protein | |
| BTH_II1713 | 7.4 | Domain of unknown function | |
| BTH_II1126 | 7.3 | DGPF domain protein | |
| BTH_II1371 | 7.2 | Protein of unknown function (DUF355) superfamily | |
| BTH_II0175 | 7.2 | hypothetical protein | |
| BTH_II0103 | 7.2 | outer membrane porin OpcP | |
| BTH_I3188 | 7.2 | glycosyl transferase, group 1 family protein | |
| BTH_I1916 | 7.2 | hypothetical protein | |
| BTH_II2194 | 7.2 | hypothetical protein | |
| BTH_I0072 | 7.1 | catalase | |
| BTH_II2069 | 7.1 | aspartate/glutamate:proton symporter, AGT family (TC 2.A.3.11.1) | |
| BTH_II1425 | 7.1 | manganese/iron transporter, NRAMP family | |
| BTH_II1215 | 7.1 | hypothetical protein | |
| BTH_II0153 | 7.0 | chemotaxis MotA protein | |
| BTH_II2139 | 7.0 | TonB-dependent heme/hemoglobin receptor family protein | |
| BTH_II2254 | 7.0 | epoxide hydrolase | |
| BTH_II0030 | 7.0 | radical SAM domain/B12 binding domain protein | |
| BTH_II1214 | 6.9 | peptide synthetase, putative | |
| BTH_II0135 | 6.9 | putative cytoplasmic protein | |
| BTH_II1216 | 6.9 | D-cysteine desulfhydrase, putative | |
| BTH_II1651 | 6.8 | amino acid/polyamine/organocation transporter, APC superfamily (TC 2.A.3) | |
| BTH_II1899 | 6.8 | Protein of unknown function (DUF796) superfamily | |
| BTH_II2111 | 6.8 | hypothetical protein | |
| BTH_II0722 | 6.8 | hypothetical protein | |
| BTH_II0251 | 6.8 | lipoprotein, putative | |
| BTH_II0386 | 6.7 | hypothetical protein | |
| BTH_II1891 | 6.7 | pentapeptide repeat family protein | |
| BTH_II1892 | 6.7 | hypothetical protein | |
| BTH_I0930 | 6.7 | hypothetical protein | |
| BTH_II2266 | 6.7 | Predicted ATPase with chaperone activity, putative | |
| BTH_II0002 | 6.6 | phage integrase family protein | |
| BTH_II0341 | 6.6 | ribosomal protein L15 | |
| BTH_II2163 | 6.6 | Amidohydrolase family superfamily | |
| BTH_II2066 | 6.6 | oxidoreductase, FAD-binding family protein | |
| BTH_II1890 | 6.6 | hypothetical protein | |
| BTH_II1634 | 6.6 | two component transcriptional regulator, LuxR family | |
| BTH_II0543 | 6.6 | hypothetical protein | |
| BTH_I1423 | 6.5 | hypothetical protein | |
| BTH_II0057 | 6.5 | hypothetical protein | |
| BTH_II1178 | 6.5 | carbohydrate ABC transporter membrane protein 1, CUT1 family (TC 3.A.1.1.-) | |
| BTH_II0249 | 6.5 | FHA domain protein | |
| BTH_II1712 | 6.5 | hypothetical protein | |
| BTH_II1009 | 6.5 | sensory box histidine kinase/response regulator | |
| BTH_I0444 | 6.5 | hypothetical protein | |
| BTH_II2253 | 6.5 | cytochrome p460 | |
| BTH_I1434 | 6.5 | activator protein, putative | |
| BTH_II0252 | 6.4 | Bacterial protein of unknown function (DUF876) superfamily | |
| BTH_II2235 | 6.4 | citrate lyase, beta subunit | |
| BTH_II0986 | 6.4 | hypothetical protein | |
| BTH_II2140 | 6.4 | hemin transport protein HmuS | |
| BTH_I2367 | 6.4 | dihydroaeruginoic acid synthetase | |
| BTH_II0280 | 6.4 | polyketide synthase, putative | |
| BTH_II0208 | 6.4 | GGDEF domain protein | |
| BTH_II1636 | 6.4 | phosphoesterase family protein | |
| BTH_II2282 | 6.4 | porin | |
| BTH_I3295 | 6.4 | 2-keto-4-methylthiobutyrate aminotransferase apoenzyme (EC 2.6.1.-) | |
| BTH_II0345 | 6.4 | hypothetical protein | |
| BTH_II1976 | 6.4 | galactoside O-acetyltransferase | |
| BTH_II0058 | 6.3 | transporter, AcrB/D/F family | |
| BTH_II0254 | 6.3 | lipoprotein, putative | |
| BTH_II0071 | 6.2 | probable hemagglutinin-related protein | |
| BTH_II1213 | 6.2 | peptide synthetase-like protein | |
| BTH_II0999 | 6.2 | sarcosine oxidase subunit gamma | |
| BTH_I1409 | 6.2 | xanthine dehydrogenase, molybdenum binding subunit apoprotein (EC 1.17.1.4) | |
| BTH_I1761 | 6.2 | amino acid/amide ABC transporter substrate-binding protein, HAAT family | |
| BTH_II2121 | 6.1 | Rieske [2Fe-2S] domain protein | |
| BTH_II0693 | 6.1 | hypothetical protein | |
| BTH_II2364 | 6.1 | CheC family protein | |
| BTH_II1007 | 6.1 | sensory box histidine kinase | |
| BTH_II0112 | 6.1 | Hep_Hag family | |
| BTH_II1217 | 6.1 | bacterial luciferase family protein | |
| BTH_I0618 | 6.0 | methyl-accepting chemotaxis sensory transducer with Pas/Pac sensor | |
| BTH_II0277 | 6.0 | Amidohydrolase family superfamily, putative | |
| BTH_II1897 | 5.9 | Bacterial protein of unknown function (DUF879) superfamily | |
| BTH_II1489 | 5.9 | Hep_Hag family | |
| BTH_II2289 | 5.9 | hypothetical protein | |
| BTH_I0332 | 5.9 | alkaline phosphatase (EC 3.1.3.1) | |
| BTH_II2230 | 5.9 | drug resistance transporter, EmrB/QacA family protein, putative | |
| BTH_I1421 | 5.9 | serine-type carboxypeptidase family protein | |
| BTH_II0164 | 5.9 | flagellar biosynthetic protein FlhB | |
| BTH_II0156 | 5.9 | chemotaxis protein CheA | |
| BTH_II0176 | 5.8 | flagellar hook-associated protein, putative | |
| BTH_II0110 | 5.8 | fusaric acid resistance protein, putative | |
| BTH_II1714 | 5.8 | Domain of unknown function | |
| BTH_II0760 | 5.8 | hypothetical protein | |
| BTH_II0942 | 5.8 | hypothetical protein | |
| BTH_II0320 | 5.8 | pyrazinamidase/nicotinamidase | |
| BTH_II1894 | 5.8 | Rhs element Vgr protein | |
| BTH_II0167 | 5.8 | flagellar biosynthetic protein fliP | |
| BTH_II2325 | 5.8 | lipoprotein, putative | |
| BTH_II0154 | 5.8 | Chemotaxis lafU protein | |
| BTH_II0763 | 5.8 | hrp protein, putative | |
| BTH_II0155 | 5.8 | chemotaxis response regulator | |
| BTH_II0565 | 5.7 | BarC | |
| BTH_I1408 | 5.7 | xanthine dehydrogenase, N-terminal subunit | |
| BTH_II1889 | 5.7 | hypothetical protein | |
| BTH_II1898 | 5.7 | hypothetical protein | |
| BTH_I0334 | 5.6 | hypothetical protein | |
| BTH_II0177 | 5.6 | flagellar protein FliS | |
| BTH_II2070 | 5.6 | 2,5-dioxopentanoate dehydrogenase (EC 1.2.1.26) | |
| BTH_II2162 | 5.6 | aldehyde dehydrogenase (NAD) family protein superfamily | |
| BTH_II0515 | 5.5 | ABC transporter, permease protein/ATP-binding protein | |
| BTH_II1888 | 5.5 | lipoprotein, putative | |
| BTH_II0179 | 5.5 | Flagellar hook-length control protein, putative | |
| BTH_II0514 | 5.5 | DJ-1/PfpI family protein | |
| BTH_II0024 | 5.5 | transcriptional regulator, PadR family | |
| BTH_II0055 | 5.5 | sulfide-quinone oxidoreductase (EC 1.8.5.4) | |
| BTH_II0340 | 5.5 | UDP-N-Acetylglucosamine 2-epimerase (EC 5.1.3.14) | |
| BTH_II1212 | 5.5 | syringomycin biosynthesis enzyme, putative | |
| BTH_II2161 | 5.4 | choline dehydrogenase | |
| BTH_II0696 | 5.4 | hypothetical protein | |
| BTH_II0171 | 5.4 | flagellar M-ring protein FliF | |
| BTH_I1688 | 5.4 | hypothetical protein | |
| BTH_II1386 | 5.4 | hypothetical protein | |
| BTH_II0546 | 5.4 | capsular polysaccharide biosynthesis/export periplasmic protein | |
| BTH_II1482 | 5.4 | hypothetical protein | |
| BTH_II1452 | 5.4 | hypothetical protein | |
| BTH_I2308 | 5.4 | hypothetical protein | |
| BTH_II1896 | 5.4 | hypothetical protein | |
| BTH_II0510 | 5.4 | hypothetical protein | |
| BTH_II0120 | 5.4 | unnamed protein product; Similar to putative exported protein | |
| BTH_I0271 | 5.4 | hypothetical protein | |
| BTH_II0137 | 5.4 | Protein of unknown function (DUF1316) subfamily, putative | |
| BTH_I2845 | 5.4 | lipoprotein, putative | |
| BTH_II1893 | 5.4 | Rhs element Vgr protein | |
| BTH_II0925 | 5.3 | beta-lactamase, putative | |
| BTH_II2082 | 5.3 | hypothetical protein | |
| BTH_II0076 | 5.3 | hypothetical protein | |
| BTH_I3107 | 5.3 | hypothetical protein | |
| BTH_II1281 | 5.3 | diaminohydroxyphosphoribosylaminopyrimidine deaminase (EC 3.5.4.26) | |
| BTH_II0563 | 5.2 | peptide synthetase, putative | |
| BTH_II2306 | 5.2 | hypothetical protein | |
| BTH_II1960 | 5.2 | copper tolerance protein | |
| BTH_II2309 | 5.2 | iron-sulfur cluster-binding protein domain protein | |
| BTH_II1895 | 5.2 | ATP-dependent Clp protease, ATP-binding subunit ClpB | |
| BTH_II0216 | 5.2 | major facilitator family transporter | |
| BTH_II0075 | 5.2 | UvrA family protein | |
| BTH_I1760 | 5.2 | oxidoreductase, FAD-binding family protein | |
| BTH_II0568 | 5.2 | BarC | |
| BTH_II0217 | 5.2 | hippurate hydolase | |
| BTH_II0077 | 5.2 | hypothetical protein | |
| BTH_II0151 | 5.1 | flagellin D | |
| BTH_I2826 | 5.1 | hypothetical protein | |
| BTH_II1451 | 5.1 | lipoprotein NlpD, putative | |
| BTH_I2315 | 5.1 | lipoprotein, putative | |
| BTH_II1003 | 5.1 | hypothetical protein | |
| BTH_II2154 | 5.1 | major facilitator family transporter | |
| BTH_II0022 | 5.1 | sperm-specific protein Phi-1 | |
| BTH_II0694 | 5.1 | polysaccharide biosynthesis family protein | |
| BTH_I1918 | 5.1 | pyocin R2_PP, tail formation | |
| BTH_II0322 | 5.1 | hypothetical protein | |
| BTH_II0021 | 5.1 | PAP2 family protein | |
| BTH_II1488 | 5.0 | Beta-barrel assembly machine subunit BamE | |
| BTH_II2083 | 5.0 | monoxygenase | |
| BTH_I1917 | 5.0 | hypothetical protein | |
| BTH_II0238 | 5.0 | pyruvate dehydrogenase E1 beta subunit | |
| BTH_II2065 | 5.0 | NAD-dependent formate dehydrogenase, alpha subunit | |
| BTH_II2074 | 5.0 | DoxD-like family protein | |
| **Locus tag** | **Fold change** | **Joint Genome Institute product name** |  |
| BTH_II1349 | -11.2 | Phage small terminase subunit |  |
| BTH_II0444 | -11.1 | ABC transporter, permease protein |  |
| BTH_I2385 | -10.2 | ornithine carbamoyltransferase |  |
| BTH_I2384 | -9.8 | arginine deiminase (EC 3.5.3.6) |  |
| BTH_II0443 | -9.3 | hypothetical protein |  |
| BTH_II0442 | -9.3 | universal stress protein family |  |
| BTH_II1054 | -9.0 | Phage tail assembly chaperone |  |
| BTH_II1350 | -9.0 | phage major capsid protein, P2 family |  |
| BTH_I1361 | -8.8 | possible phosphatase |  |
| BTH_I1356 | -8.8 | Capsule polysaccharide biosynthesis protein family |  |
| BTH_II2252 | -8.7 | carbon starvation protein A |  |
| BTH_I1360 | -8.2 | 3-deoxy-8-phosphooctulonate synthase |  |
| BTH_I1358 | -8.0 | putative glycosyltransferase |  |
| BTH_I1359 | -8.0 | glycosyltransferase |  |
| BTH_I1362 | -7.9 | carbohydrate isomerase, KpsF/GutQ family |  |
| BTH_II1048 | -7.8 | hypothetical protein |  |
| BTH_I0599 | -7.7 | glycerol kinase (EC 2.7.1.30) |  |
| BTH_I1354 | -7.4 | exopolysaccharide tyrosine-protein kinase, putative |  |
| BTH_I1357 | -7.4 | satase isoform II |  |
| BTH_II1052 | -7.3 | gp10 |  |
| BTH_I2383 | -7.2 | arginine:ornithine antiporter, APA family (TC 2.A.3.2.3) |  |
| BTH_I1363 | -7.1 | UDP-glucose pyrophosphorylase (EC 2.7.7.9) |  |
| BTH_II1053 | -7.0 | gp11 |  |
| BTH_I2386 | -6.9 | carbamate kinase |  |
| BTH_I0598 | -6.9 | glycerol uptake facilitator protein |  |
| BTH_I1353 | -6.5 | low molecular weight protein-tyrosine-phosphatase |  |
| BTH_II1049 | -6.5 | gp7 |  |
| BTH_II0884 | -6.5 | hypothetical protein |  |
| BTH_I0243 | -6.5 | flagellar hook protein FlgE |  |
| BTH_II1344 | -6.5 | Protein of unknown function (DUF754) superfamily |  |
| BTH_II1351 | -6.5 | Phage capsid scaffolding protein (GPO) |  |
| BTH_I0245 | -6.3 | flagellar basal-body rod protein FlgG |  |
| BTH_I0241 | -6.3 | flagellar basal-body rod protein FlgC |  |
| BTH_I1801 | -6.0 | cyclic nucleotide-binding domain protein |  |
| BTH_II0883 | -6.0 | hypothetical protein |  |
| BTH_I2722 | -5.9 | hypothetical protein |  |
| BTH_I1352 | -5.9 | capsular polysaccharide biosynthesis/export periplasmic protein |  |
| BTH_I0242 | -5.7 | basal-body rod modification protein FlgD |  |
| BTH_II1057 | -5.6 | Phage minor tail protein |  |
| BTH_I0244 | -5.6 | flagellar basal-body rod protein FlgF |  |
| BTH_I1800 | -5.5 | oxygen-independent coproporphyrinogen III oxidase |  |
| BTH_II0985 | -5.5 | Bacterial extracellular solute-binding protein, family 7 superfamily |  |
| BTH_II1051 | -5.4 | phage protein, HK97 gp10 family |  |
| BTH_II0445 | -5.4 | ABC transporter, ATP-binding protein |  |
| BTH_II1055 | -5.3 | gp13 |  |
| BTH_II0882 | -5.3 | hypothetical protein |  |
| BTH_II1331 | -5.3 | Phage tail sheath protein |  |
| BTH_II1347 | -5.3 | hypothetical protein |  |
| BTH_I2273 | -5.3 | outer membrane protein, OmpW family |  |
| BTH_I1355 | -5.3 | hypothetical protein |  |
| BTH_I1515 | -5.2 | hypothetical protein |  |
| BTH_II1835 | -5.2 | L-leucine/L-isoleucine/L-valine ABC transporter ATP-binding protein |  |
| BTH_II1060 | -5.1 | gp19 |  |
| BTH_I0031 | -5.1 | flagellar biosynthetic protein FliQ |  |
| BTH_II1047 | -5.1 | phage major capsid protein, HK97 family |  |
| BTH_I1849 | -5.1 | two component transcriptional regulator, LuxR family |  |
| BTH_II1345 | -5.1 | hypothetical protein |  |
| BTH_II1050 | -5.1 | phage head-tail adaptor, putative |  |
| BTH_II1836 | -5.1 | L-leucine/L-isoleucine/L-valine ABC transporter ATP-binding protein |  |
| BTH_I3206 | -5.0 | curli production assembly/transport component CsgG, putative |  |
| BTH_I0028 | -5.0 | flagellar motor switch protein FliN |  |
| BTH_I2480 | -5.0 | sulfate ABC transporter, periplasmic sulfate-binding protein |  |
| BTH_I3211 | -5.0 | aminotransferase, DegT/DnrJ/EryC1/StrS family |  |
