# Supplementary material for: Multi-Omic Analyses Provide Links between Low-Dose Antibiotic Treatment and Induction of Secondary Metabolism in Burkholderia thailandensis
Source: mBio. 2020 Feb 25;11(1):e03210-19. doi: 10.1128/mBio.03210-19 (PMC7042699; doi:10.1128/mBio.03210-19)
Supplement: TABLE S3 [file mBio.03210-19-st003.docx]

**Table S3** Top 25 most up-regulated proteins (top) and most down-regulated proteins (bottom) as determined by TMTc+ at OD_600_ ~1.0. The averages of four independent measurements are shown. Standard errors were typically <10% of the mean values reported.

| **Locus tag** | **log2 fold-change** | **Predicted function** |
| --- | --- | --- |
| BTH_II2089 | 6.6 | hypothetical protein |
| BTH_II1703 | 6.2 | uncharacterized domain protein |
| BTH_I0702 | 5.9 | xylulokinase |
| BTH_II1314 | 5.7 | hypothetical protein |
| BTH_I2949 | 5.6 | colicin V processing peptidase. Cysteine peptidase. MEROPS family C39 |
| BTH_I2945 | 5.2 | peptidase, M1 family |
| BTH_II2090 | 5.1 | syringomycin synthesis regulator SyrP, putative |
| BTH_II0407 | 5.1 | glutathione S-transferase |
| BTH_I0234 | 4.9 | NADH-ubiquinone oxidoreductase, putative |
| BTH_II2094 | 4.8 | ketol-acid reductoisomerase (EC 1.1.1.86) |
| BTH_II0570 | 4.7 | 4-hydroxy-2-oxovalerate aldolase |
| BTH_II1714 | 4.7 | Domain of unknown function |
| BTH_II1900 | 4.6 | Protein of unknown function (DUF877) |
| BTH_II2096 | 4.6 | long-chain-fatty-acid--CoA ligase, putative |
| BTH_II0151 | 4.6 | flagellin D |
| BTH_I1606 | 4.5 | methionine synthase (B12-independent) (EC 2.1.1.14) |
| BTH_I2669 | 4.5 | hypothetical protein |
| BTH_II2105 | 4.5 | hydrophobe/amphiphile efflux family protein |
| BTH_II2098 | 4.4 | malonyl CoA-acyl carrier protein transacylase |
| BTH_II1899 | 4.4 | Protein of unknown function (DUF796) superfamily |
| BTH_I1263 | 4.4 | hypothetical protein |
| BTH_I2365 | 4.4 | polyketide synthase |
| BTH_I2367 | 4.3 | dihydroaeruginoic acid synthetase |
| BTH_II1713 | 4.3 | Domain of unknown function |
| BTH_I0450 | 4.3 | PTS system N-acetylglucosamine-specific IIB/IIC component, Glc family (TC 4.A.1.1.2)/(TC 4.A.1.1.2) |
| **Locus tag** | **log2 fold-change** | **Predicted function** |
| BTH_I0786 | -6.6 | amino acid/amide ABC transporter membrane protein 2, HAAT family (TC 3.A.1.4.-) |
| BTH_I1787 | -6.1 | cytochrome bo3 quinol oxidase subunit 1 apoprotein (EC 1.10.3.-) |
| BTH_I2541 | -4.2 | hypothetical protein |
| BTH_I2450 | -3.2 | amino acid ABC transporter substrate-binding protein, PAAT family (TC 3.A.1.3.-) |
| BTH_II2316 | -3.1 | sulfate permease family protein |
| BTH_II0800 | -3.1 | taurine ABC transporter, periplasmic binding protein |
| BTH_II0834 | -3.0 | BsaU protein |
| BTH_I2723 | -2.9 | filamentous haemagglutinin |
| BTH_I0338 | -2.5 | dethiobiotin synthetase |
| BTH_I2477 | -2.4 | sulfate ABC transporter, ATP-binding protein |
| BTH_I3204 | -2.1 | lipoprotein, putative |
| BTH_I2722 | -2.1 | hypothetical protein |
| BTH_II0783 | -2.1 | hypothetical protein |
| BTH_I2480 | -2.0 | sulfate ABC transporter, periplasmic sulfate-binding protein |
| BTH_II0638 | -1.9 | TonB-dependent copper receptor |
| BTH_I2476 | -1.9 | cys regulon transcriptional activator |
| BTH_II0829 | -1.9 | type III secretion system protein BsaP |
| BTH_I1515 | -1.9 | hypothetical protein |
| BTH_I0305 | -1.8 | outer membrane porin |
| BTH_II1469 | -1.8 | aliphatic compound ABC transporter, periplasmic substrate-binding protein |
| BTH_II0835 | -1.8 | type III secretion system protein BsaV |
| BTH_II1202 | -1.7 | hypothetical protein |
| BTH_II1227 | -1.7 | N-acyl homoserine lactone synthase |
| BTH_I1846 | -1.7 | nitrogen regulation protein NR(I) |
| BTH_II0832 | -1.7 | type III secretion system protein BsaS |
