# Supplementary material for: Multi-Omic Analyses Provide Links between Low-Dose Antibiotic Treatment and Induction of Secondary Metabolism in Burkholderia thailandensis
Source: mBio. 2020 Feb 25;11(1):e03210-19. doi: 10.1128/mBio.03210-19 (PMC7042699; doi:10.1128/mBio.03210-19)
Supplement: TABLE S4 [file mBio.03210-19-st004.docx]

**Table S4**  Top 25 most up-regulated proteins (top) and down-regulated proteins (bottom) by TMTc+ at OD_600_ ~5.0. The averages of four independent measurements are shown. Standard errors were typically <10% of the mean values reported.

| **Locus tag** | | **log2 fold-change** | **Predicted function** |  |
| --- | --- | --- | --- | --- |
| BTH_II0151 | | 8.1 | flagellin D |  |
| BTH_II2160 | | 6.9 | transcriptional regulator, LysR family |  |
| BTH_I1606 | | 5.9 | methionine synthase (B12-independent) (EC 2.1.1.14) |  |
| BTH_II2105 | | 5.3 | hydrophobe/amphiphile efflux family protein |  |
| BTH_I2945 | | 5.1 | peptidase, M1 family |  |
| BTH_I1999 | | 5.0 | ATP-dependent RNA helicase RhlE |  |
| BTH_II2104 | | 4.7 | RND efflux system, outer membrane lipoprotein, NodT family |  |
| BTH_II1931 | | 4.5 | metallo-beta-lactamase superfamily domain protein |  |
| BTH_II1727 | | 4.5 | ABC transporter, ATP-binding protein |  |
| BTH_I0524 | | 4.4 | glycosyl transferase, group 1 family protein |  |
| BTH_II2348 | | 4.3 | polyketide synthase, putative |  |
| BTH_II2107 | | 4.2 | lipH |  |
| BTH_II0204 | | 4.2 | peptide synthetase, putative |  |
| BTH_II2089 | | 4.2 | hypothetical protein |  |
| BTH_I0522 | | 4.1 | mannose-1-phosphate guanylyltransferase (GDP) (EC 2.7.7.22)/mannose-6-phosphate isomerase, type 2 (EC 5.3.1.8) |  |
| BTH_I1263 | | 4.1 | hypothetical protein |  |
| BTH_II1752 | | 4.1 | Trehalase |  |
| BTH_II1932 | | 4.0 | 3-oxoacyl-(acyl carrier protein) synthase III |  |
| BTH_II1663 | | 3.9 | pyruvate ferredoxin/flavodoxin oxidoreductase |  |
| BTH_II1664 | | 3.9 | polyketide synthase, putative |  |
| BTH_II0206 | | 3.6 | hypothetical protein |  |
| BTH_II1665 | | 3.6 | polyketide synthase, putative |  |
| BTH_II1666 | | 3.4 | polyketide synthase, putative |  |
| BTH_II1935 | | 3.4 | acetyl-CoA synthetase, putative |  |
| BTH_II1930 | | 3.4 | AMP-binding domain protein |  |
| **Locus tag** | **log2 fold-change** | | **Predicted function** | |
| BTH_II0258 | -13.3 | | Protein of unknown function (DUF770) superfamily | |
| BTH_II0649 | -13.2 | | Tyrosinase (Monophenol monooxygenase) | |
| BTH_I1714 | -13.2 | | Uncharacterized ACR, COG1399 | |
| BTH_I1995 | -12.2 | | haloacid dehalogenase, type II | |
| BTH_II2220 | -11.6 | | transcriptional regulator, XRE family with cupin sensor | |
| BTH_I1853 | -8.1 | | respiratory nitrate reductase beta subunit | |
| BTH_I2339 | -7.4 | | xylose-binding protein | |
| BTH_I2673 | -6.7 | | trans-aconitate methyltransferase | |
| BTH_II1418 | -5.8 | | magnesium-translocating P-type ATPase | |
| BTH_I1854 | -5.8 | | respiratory nitrate reductase alpha subunit apoprotein | |
| BTH_II1419 | -5.4 | | hypothetical protein | |
| BTH_I2307 | -5.3 | | hypothetical protein | |
| BTH_I2273 | -5.3 | | outer membrane protein, OmpW family | |
| BTH_I2383 | -4.9 | | arginine:ornithine antiporter, APA family (TC 2.A.3.2.3) | |
| BTH_I2601 | -4.7 | | putative flavoprotein reductase | |
| BTH_II0027 | -4.5 | | hypothetical protein | |
| BTH_I0267 | -4.2 | | porin, OprB family (TC 1.B.19) | |
| BTH_II2026 | -4.0 | | outer membrane transport energization protein ExbD (TC 2.C.1.1.1) | |
| BTH_I1997 | -4.1 | | universal stress protein family | |
| BTH_II0442 | -4.0 | | universal stress protein family | |
| BTH_I0511 | -3.9 | | hypothetical protein | |
| BTH_I0866 | -3.8 | | hypothetical protein | |
| BTH_II1774 | -3.8 | | serine protease, subtilase family | |
| BTH_II2121 | -3.7 | | Rieske [2Fe-2S] domain protein | |
| BTH_I2334 | -3.6 | | hypothetical protein | |
