# Supplementary material for: Multi-Omic Analyses Provide Links between Low-Dose Antibiotic Treatment and Induction of Secondary Metabolism in Burkholderia thailandensis
Source: mBio. 2020 Feb 25;11(1):e03210-19. doi: 10.1128/mBio.03210-19 (PMC7042699; doi:10.1128/mBio.03210-19)
Supplement: TABLE S5 [file mBio.03210-19-st005.docx]

**Table S5:** Primers used in this study.

| **Primer name** | **Primer sequence** | **Purpose** |
| --- | --- | --- |
| ncRelA-UpF | AAC GAC ATC GGG CCG CAC ATC | Construction of *kan^R^* insertional *relA* deletion mutant |
| ncRelA-UpR | GGG GTT CCG CGC ACA TTT CCC CGA AAG AAC GCG AGC ACG TCG TCA AG |  |
| ncRelA-DnF | CGC CTT CTA TCG CCT TCT TGA CGA GTT CTT CTG AAA CGG CTT GCG CAT TTC CCC C |  |
| ncRelA-DnR | TGC TCG TTC TTC GAA TCG CCG C |  |
| proKan-Fw | TTT CGG GGA AAT GTG CGC GGA AC |  |
| proKan-Rv | TCA GAA GAA CTC GTC AAG AAG GCG ATA G |  |
| ncSpoT-UpF | CTG GAG TAA CGC AAC ATG ACC GAT TC | Constrution of *tet^R^* insertional *spoT* deletion mutant |
| ncSpoT-UpR | CAA ATA GGG GTT CCG CGC ACA TTT CCT TTC GCC TCC TTG GTT CGC TCG |  |
| ncSpoT-DnF | GTG CAT GGA GCC GGG CCA CCT CGA CCT GATGC GCG TGC GGC GCC C |  |
| ncSpoT-DnR | TCC AGC TTA CAT GCC CGC GAT C |  |
| proTet-Fw | GGA AAT GTG CGC GGA ACC CC |  |
| proTet-Rv | TCA GGT CGA GGT GGC CCG G |  |
| ncRecA-UpF | TCG TCC GGC GGC GCG AAC | Constrution of *tet^R^* insertional *recA* deletion mutant |
| ncRecA-UpR | CAA ATA GGG GTT CCG CGC ACA TTT CCG AAT CGT CCT TTG CTA TGA TGA GCA GC |  |
| ncRecA-DnF | GTG CAT GGA GCC GGG CCA CCT CGA CCT GAT GCG CAA GAA CCG GCA GGG C |  |
| ncRecA-DnR | ATG GAG ACC AAT CAG CGA AAT CAC GG |  |
| RecA_UpF_HindIII | ATC TAT AAG CTT TCG TCC GGC GGC GCG AAC | Construction of markerless *recA* deletion mutant |
| RecA_UpR | GCC CTG CCG GTT CTT GCG CAT GAA TCG TCC TTT GCT ATG ATG AGC AGC |  |
| RecA_DnF | GCT GCT CAT CAT AGC AAA GGA CGA TTC ATG CGC AAG AAC CGG CAG GGC |  |
| RecA_DnR_XbaI | ATC TAT TCT AGA ATG GAG ACC AAT CAG CGA AAT CAC GG |  |
| MalR_UpF | CCT GCA GGT CGA CTC TAG AGG ATG GCC CGA CAG AAA GAC | Construction of markerless *malR* deletion mutant |
| MalR_UpR | GGC GCC CGC TCT GCG CAC TCC GGC G |  |
| MalR_DnF | GAG TGC GCA GAG CGG GCG CCG CC |  |
| MalR_DnR | TAC GAA TTC GAG CTC GGT ACA AAG ATC CCG TCG AAC TTG CC |  |
| MetZ_UpF | GCA TCG GCT TCG AGT TCG TCC | Construction of *tet^R^* insertional *metZ* deletion mutant |
| MetZ_UpR | GGA GGT TCC TGT GCG GAG CAG CTA TCG ACT C |  |
| MetZ_DnF | CAC CAC TCC CAC GCG CTT GCT GCT AAG CTG |  |
| MetZ_DnR | CGA TCG AAG TGC TCG ACG AAT CC |  |
| Tet_Fw1 | CTG CTC CGC ACA GGA ACC TCC TTT AAC TCC TCA AAA CG |  |
| Tet_Rv1 | GCA AGC GCG TGG GAG TGG TGA ATC CGT TAG CGA GG |  |
| MetH_UpF | AGC AGC GCA TCC TGA TCC TCG | Construction of *tet^R^* insertional *metH* deletion mutant |
| MetH_UpR | GCT GAT GTT CGT GCG TGC GTA TTC GGG TTG GAG |  |
| MetH_DnF | CGG ATT CAC CTG CCT GAG AGC CGA CGA ACG |  |
| MetH_DnR | GGA CAC GAA TTC GAG CAG CAC G |  |
| Tet_Fw2 | GAA TAC GCA CGC ACG AAC ATC AGC GGT CCG GGC |  |
| Tet_Rv2 | GCT CTC AGG CAG GTG AAT CCG TTA GCG AGG TGC |  |
| ncMftR_UpF | CCG ACG AAC AGC GAC GCG ATC |  |
| ncMftR_UpR | TTC CCC GAA ACC ATT AAC CCT CCT GCT CGT CCA A |  |
| ncMftR_DnF | AGT TCT TCT GAG CTG ACC GTT AGG CGT CGC G | Construction of *kant^R^* insertional *mftR*  deletion mutant |
| ncMftR_DnR | CAG CTC TAT TCG CTG CCG ACG |  |
| ncMftR_KanF | GGG TTA ATG GTT TCG GGG AAA TGT GCG CGG AAC |  |
| ncMftR_KanR | ACG GTC AGC TCA GAA GAA CTC GTC AAG AAG GCG ATA G |  |
| btaI1_rt_Fw | ATG CGA ACT TTC GTT CAT GGC GAC G | RT-qPCR primers |
| btaI1_rt_Rv | TTG CCG ACG GCA GTT TCC AGC |  |
| btaI2_rt_Fw | CCA CCG TCA AGG CAG CAC TCG |  |
| btaI2_rt_Rv | GAT CGT ATC GGG GCG GTC GAA TTG |  |
| btaI3_rt_Fw | TTG CGA ACG CCG CCG CCG |  |
| btaI3_rt_Rv | GTG TGC GGT TTC GAA GGC TGT G |  |
| btaR1_rt_Fw | ATG GAA CTG CGC TGG CAA GAC G |  |
| btaR1_rt_Rv | CAG CAA TAT TCG AAG CCG AGC CG |  |
| btaR2_rt_Fw | ATG CAC GAT TTT CTT CAA TTT TGG CTA AAC G |  |
| btaR2_rt_Rv | GTA TTC GTA GCC CAG GGT GGC |  |
| btaR3_rt_Fw | CGC ACG CTG ATC GAG ACT TTC AGG |  |
| btaR3_rt_Rv | CGG ATA GTG CAG CGA GAC GAC G |  |
| btaR4_rt_Fw | GTG CGT CGC GTG TGC GGC |  |
| btaR4_rt_Rv | ACC GAC GAT CGA ACA GGA CAT GC |  |
| btaR5_rt_Fw | ATG AGG GCG GCG ATG GGG AAT TG |  |
| btaR5_rt_Rv | ACG CGC AGT ATT CGA AAT CGA GGG |  |
| scmR_rt_Fw | ATG AAC CAA ATC CAG ACC ATG CGT GTC |  |
| scmR_rt_Rv | ATG GAT GAG ACG CGT GTT CAG ATG TG |  |
